# Integrating transcriptome-wide study and mRNA expression profiles yields novel insights into the biological mechanism of chondropathies

**DOI:** 10.1186/s13075-019-1978-8

**Published:** 2019-08-27

**Authors:** Ping Li, Yujie Ning, Xiong Guo, Yan Wen, Bolun Cheng, Mei Ma, Lu Zhang, Shiqiang Cheng, Sen Wang, Feng Zhang

**Affiliations:** Key Laboratory of Trace Elements and Endemic Diseases of National Health and Family Planning Commission, Key Laboratory of Environment and Genes Related to Diseases of Ministry of Education, Collaborative Innovation Center of Endemic Diseases and Health Promotion in Silk Road Region, School of Public Health, Health Science Center, Xi’an Jiaotong University, No.76 Yan Ta West Road, Xi’an, 710061 Shaanxi People’s Republic of China

**Keywords:** Chondropathies, Genome-wide association studies (GWAS), Transcriptome-wide association study (TWAS), mRNA expression profile

## Abstract

**Background:**

Chondropathies are a group of cartilage diseases, which share some common pathogenetic features. The etiology of chondropathies is still largely obscure now.

**Methods:**

A transcriptome-wide association study (TWAS) was performed using the UK Biobank genome-wide association study (GWAS) data of chondropathies (including 1314 chondropathy patients and 450,950 controls) with gene expression references of muscle skeleton (MS) and peripheral blood (YBL). The candidate genes identified by TWAS were further compared with three gene expression profiles of osteoarthritis (OA), cartilage tumor (CT), and spinal disc herniation (SDH), to confirm the functional relevance between the chondropathies and the candidate genes identified by TWAS. Functional mapping and annotation (FUMA) was used for the gene ontology enrichment analyses. Immunohistochemistry (IHC) was conducted to validate the accuracy of integrative analysis results.

**Results:**

Integrating TWAS and mRNA expression profiles detected 84 candidate genes for knee OA, such as DDX20 (*P*_TWAS YBL_ = 1.79 × 10^− 3^, fold change (FC) = 2.69), 10 candidate genes for CT, such as SRGN (*P*_TWAS YBL_ = 1.46 × 10^− 3^, FC = 3.36), and 4 candidate genes for SDH, such as SUPV3L1 (*P*_TWAS YBL_ = 3.59 × 10^− 3^, FC = 3.22). Gene set enrichment analysis detected 73 GO terms for knee OA, 3 GO terms for CT, and 1 GO term for SDH, such as mitochondrial protein complex (*P* = 7.31 × 10^− 5^) for knee OA, cytokine for CT (*P* = 1.13 × 10^− 4^), and ion binding for SDH (*P* = 3.55 × 10^− 4^). IHC confirmed that the protein expression level of DDX20 was significantly different between knee OA cartilage and healthy control cartilage (*P* = 0.0358).

**Conclusions:**

Multiple candidate genes and GO terms were detected for chondropathies. Our findings may provide a novel insight in the molecular mechanisms of chondropathies.

**Electronic supplementary material:**

The online version of this article (10.1186/s13075-019-1978-8) contains supplementary material, which is available to authorized users.

## Introduction

Chondropathies are a group of cartilage diseases that deviate from or interrupt the normal structure and function of cartilage, including osteoarthritis (OA), achondroplasia, spinal disc herniation (SDH), relapsing polychondritis, cartilage tumor (CT), and chondrocalcinosis [1]. OA is the most well-known chondropathy in the world, affecting the health of approximately 15% of the population, especially people older than 60 years [2]. The prevalence of disc herniation, counting in the people who suffer from low back pain, is reported variable, approximately from 0 to 47% [3]. The biological mechanism of chondropathies remains largely unknown, and there is no effective way to treat the cartilage damage recently.

Risk factors for chondropathies mainly include trauma, genetics, age, sex, obesity, and degenerative pathology [2]. Recent studies demonstrated the implication of genetic factors in the development of chondropathies. For example, GDF5 was identified as a bone morphogenetic protein involved in joint formation [4]. The mice experiment has demonstrated the cooperation of Gli2 and p53 genes involved in the regulation of chondrocyte apoptosis in malignant cartilage tumors [5]. In addition, family and twin studies have detected that a large amount of genetic factors may be responsible for sciatica, disc herniation, and disc degeneration, and two collagen IX alleles have been identified as significant loci for sciatica and lumbar disc herniation [6].

Due to the non-vascular form, damaged cartilage has limited intrinsic capacity to repair and usually leads to tissue deterioration [7]. Another character of cartilage is its non-innervated form, which means cartilage damage shows asymptomatic in the early stage and becomes symptomatic with pain until it involves the adjacent innervated tissue [8]. These are the common processes shared by various chondropathies. Moreover, cartilage matrix degradation, chondrocyte death, inflammation, and mitochondrial dysfunction have been demonstrated as the homologous features in more than two types of chondropathies. For instance, inflammation is involved in OA, relapsing polychondritis, and SDH. Cartilage matrix degradation could be found in most types of chondropathies [9]. Moreover, some common causal genes have been demonstrated in different types of chondropathies, such as tumor necrosis factor α (TNF-α) and interleukin 1β (IL-1β) [10]. However, limited efforts have been made to explore the common biological mechanism of various chondropathies. Most previous studies only focused on one type of chondropathies, such as knee OA, hip OA, CT, or SDH, which masked the homologous features for all the chondropathies.

Over the past few years, genome-wide association studies (GWAS) have identified significant genetic variants for chondropathies [11]. However, GWAS-identified variants are located in non-coding regions, which could hardly explain the relative risk [12]. The genes contributing to these associations remain largely unknown. Recently, integrative analysis of GWAS and non-coding genetic regulatory loci information has attracted more attention. A powerful approach called transcriptome-wide association study (TWAS) was proposed to identify expression-trait based causal genes by integrating gene expression data with GWAS data [13]. The TWAS, based on the GWAS data and gene expression imputation, shows effectively in identifying the causal genes whose expression are related to human complex traits and diseases [12].

In this study, TWAS was adopted from a large-scale GWAS of chondropathies to identify mRNA expression associated genes for chondropathies. Next, the candidate genes identified by TWAS were compared with the mRNA expression profiles of OA, CT, and SDH, to further confirm the functional relevance of identified candidate genes with chondropathies. The overlapped common genes shared by TWAS and mRNA expression profiles were subjected to functional enrichment analyses.

## Methods

### The GWAS summary data

The GWAS summary data of chondropathies were obtained from the UK Biobank (http://geneatlas.roslin.ed.ac.uk/). All study subjects were of European ancestry, including 1314 chondropathy patients and 450,950 controls, aged between 40 and 69 years (UK Biobank Field: 41202, 41204) [14, 15]. The UK Biobank is a large prospective epidemiological study, recruiting nearly 500,000 deeply phenotyped individuals [14]. Briefly, DNA was extracted from blood samples stored at the UK Biobank facility for genotyping. Both marker-based quality control and sample-based quality control procedures were implemented by principal component analysis accounting for possible population structure in the study samples [14]. Genome-wide genotyping was conducted using either the Affymetrix UK BiLEVE Axiom or Affymetrix UK Biobank Axiom array. Linear mixed model was used for genome-wide SNP association testing. Detailed information of study samples, study design, and statistical analysis could be found in the published study [14, 15].

### Gene expression profiles of knee OA

The mRNA expression profiles of knee OA cartilage tissue were obtained from a previous study [16]. The knee cartilage specimens were collected from 6 knee OA patients (1 male/5 females) and 5 normal control (3 males/2 females). Each cartilage specimen was disserted into two groups, one for microarray experiment and the other for quantitative reverse transcription polymerase chain reaction (qRT-PCR) validation. The extracted mRNA was purified, amplified, and transcribed into fluorescent cRNA after removing rRNA from the total RNA. The quality of cRNAs was measured using the Nanodrop ND-1000. Then, cRNA was hybridized to the Human lncRNA Array following the Agilent One-Color Microarray-Based Gene Expression Analysis protocol (Agilent Technology). Significant regulated mRNAs were defined as fold change (FC) ≥ 2.0 and *P* value < 0.05. Detailed information of study samples, study design, and statistical analysis could be found in Additional file 1: Table S1 and the published study [16].

### Gene expression profiles of CT

The mRNA expression profiles of CT were obtained from the GEO database (https://www.ncbi.nlm.nih.gov/geo/) (Access number: GSE22855) [17]. Ollier disease is a rare disorder and develops multiple benign cartilage tumors called enchondromas [17]. This dataset contained 7 Ollier enchondrama patients as well as 2 growth plates and 4 articular cartilage served as controls [17]. Total RNA was isolated from the fresh frozen tissue and measured by an RNA 600 Nano LabChip kit with Agilent 2100 Bioanalyzer (Santa Clara, CA, USA). Genome-wide mRNA expression profiling was conducted using the Illumina BeadArray v3.0 Chip. The processed expression profile data was analyzed by the linear models for microarray (LIMMA) tool. Detailed information of study samples, study design, and statistical analysis could be found in Additional file 1: Table S1 and the published study [17].

### Gene expression profiles of SDH

The significantly differently expressed genes in SDH were obtained from a published study [18]. Briefly, intervertebral disc (IVD) tissue specimens were collected from 10 degenerative disc patients (6 females/4 males, 21–43 years) and 10 healthy controls (5 females/5 males, 28–55 years). Total RNA was isolated from whole IVD tissue, reverse-transcribed into cRNA, and hybridized to Agilent Human 1A microarray chip. Microarrays were scanned by Gene-Pix 4000B and analyzed by GenePixPro 3.0 (Axon Instruments, Inc., CA, USA). The genes were considered significantly differentially expressed when *P* value < 0.05 and FC ≥ 2.0. Detailed information of study samples, study design, and statistical analysis could be found in Additional file 1: Table S1 and the published study [18].

### Immunohistochemistry (IHC)

Two genes (CSF1R and DDX20) of the top 10 candidate genes of knee OA were randomly selected for IHC. Knee cartilage specimens were collected from 5 knee OA patients and 4 heathy controls (Additional file 2: Table S2). Healthy knee specimens were collected from the subjects undergoing amputation caused by traffic accident. Primary knee OA was diagnosed according to careful clinical and radiography examination. Subjects with genetic bone, cartilage, and other skeletal disorders were excluded from this study. For IHC, the cartilage specimens were prepared and embedded in paraffin. Then, deparaffinization and rehydration by citrate buffer (pH 6.0) overnight at 37 °C and 12.5% trypsin (Xi’an GuoAn Biological Technology Co.) at 37 °C for 20–30 min were done. After that, Zhong Shan Gold Bridge Rabbit SP reagent was used in the following procession (Beijing Zhong Gold Bridge Biological Technology Co., 18112A11) according to the manual: 3% hydrogen peroxide solution for 10 min at room temperature (RT) (white bottle), blocking in serum for 20 min at RT (blue bottle), primary antibody of CSF1R (1:50, Proteintech) and DDX20 (1:50, Proteintech) incubated overnight at 4 °C, secondary antibody for 18 min at 37 °C (yellow bottle), and horseradish peroxidase (HRP) for 18 min at 37 °C (red bottle).

### Statistical analysis

The TWAS of chondropathies was performed by the FUSION software (http://gusevlab.org/projects/fusion/) [13]. Using pre-computed gene expression weights together with GWAS summary data, FUSION is capable of evaluating the gene expression associations between every gene and target diseases. In this work, TWAS analysis was performed based on the chondropathies GWAS dataset obtained from the UK Biobank as well as gene expression references of muscle skeleton (MS) and peripheral blood (YBL) from FUSION [13, 15]. For mRNA expression profile analysis, the differentially expressed genes of knee OA and SDH were identified using an unpaired *t*-test. The mRNA expression profile analysis of CT (Ollier disease) was performed using the GEO2R tool of GEO [19]. The candidate genes identified by TWAS were compared with the significant expressed genes detected by mRNA expression profiles to detect common genes. GO enrichment analyses of the identified common genes was performed by the functional mapping and annotation (FUMA) software [20]. For IHC data analysis, ImageJ was used to analyze the percentage of positive chondrocytes and *t*-test was conducted using SPSS 19.0.

## Results

### TWAS results of chondropathies

TWAS of chondropathies identified 195 genes in MS and 252 genes in YBL with *P*_TWAS_ < 0.05 (Additional file 3: Table S3, and Additional file 4: Table S4). For instance, NSA2 (*P*_TWAS MS_ = 2.47 × 10^− 4^), TP53I13 (*P*_TWAS MS_ = 2.63 × 10^− 4^), and FPGT (*P*_TWAS MS_ = 3.69 × 10^− 4^) were the top three significant genes in MS, and RSRC1 (*P*_TWAS YBL_ = 1.03 × 10^− 4^), NSA2 (*P*_TWAS YBL_ = 3.30 × 10^− 4^), and TPD52 (*P*_TWAS YBL_ = 3.62 × 10^− 4^) were the top three significant genes in YBL.

We also detected 20 overlapped genes in both MS and YBL (Table 1) in chondropathies, such as NSA2 (*P*_TWAS MS_ = 2.47 × 10^− 4^, *P*_TWAS YBL_ = 3.30 × 10^− 4^), RSRC1 (*P*_TWAS MS_ = 4.54 × 10^− 4^, *P*_TWAS YBL_ = 1.03 × 10^− 4^), NME6 (*P*_TWAS MS_ = 9.93 × 10^− 3^, *P*_TWAS YBL_ = 1.75 × 10^− 3^), NUDT2 (*P*_TWAS MS_ = 1.85 × 10^− 2^, *P*_TWAS YBL_ = 2.32 × 10^− 2^), and ACADM (*P*_TWAS MS_ = 3.03 × 10^− 2^, *P*_TWAS YBL_ = 4.14 × 10^− 2^).

**Table 1:** List of 20 overlapped genes identified by TWAS in both MS and YBL for chondropathies

Gene	Chromosome	*P*_TWAS MS_	*P*_TWAS YBL_
ACADM	1	3.03 × 10^− 2^	4.14 × 10^− 2^
ADIPOR1	1	3.83 × 10^− 2^	9.15 × 10^− 3^
AGA	4	4.34 × 10^− 2^	1.48 × 10^− 2^
ARMC1	8	1.62 × 10^− 3^	8.76 × 10^− 3^
CHURC1	14	1.59 × 10^− 2^	1.63 × 10^− 2^
FAM149B1	10	5.03 × 10^−3^	5.30 × 10^− 3^
FPGT	1	3.69 × 10^−4^	1.40 × 10^− 2^
KHK	2	1.57 × 10^−2^	1.02 × 10^− 2^
MTFR1	8	5.32 × 10^−3^	3.30 × 10^−2^
NDFIP1	5	7.48 × 10^−3^	3.82 × 10^−2^
NDUFA10	2	1.56 × 10^−2^	6.11 × 10^−3^
NME6	3	9.93 × 10^−3^	1.75 × 10^−3^
NSA2	5	2.47 × 10^−4^	3.30 × 10^−4^
NUDT13	10	9.46 × 10^−3^	2.77 × 10^− 3^
NUDT2	9	1.85 × 10^−2^	2.32 × 10^− 2^
PHLPP2	16	7.58 × 10^−3^	1.88 × 10^−2^
RSRC1	3	4.54 × 10^−4^	1.03 × 10^− 4^
SH3GLB2	9	1.46 × 10^−2^	7.00 × 10^−3^
TAF1B	2	2.85 × 10^−2^	2.38 × 10^−2^
TRIM66	11	2.80 × 10^−2^	2.86 × 10^−2^

Note: *P*_TWAS MS_ and *P*_TWAS YBL_ denote the TWAS *P* value in muscle skeleton (MS) and peripheral blood (YBL)

### Integrative analysis of TWAS and mRNA expression profiles

The top 10 candidate genes identified for knee OA, CT, and SDH are listed in Table 2. For knee OA, we detected 84 overlapped genes shared by TWAS and mRNA expression profiling (Additional file 5: Table S5), such as NSA2 (*P*_TWAS MS_ = 2.47 × 10^− 4^, *P*_TWAS YBL_ = 3.30 × 10^− 4^, mRNA FC = − 2.16), CSF1R (*P*_TWAS YBL_ = 3.95 × 10^− 4^, mRNA FC = 3.47), MSC (*P*_TWAS YBL_ = 9.51 × 10^− 4^, mRNA FC = 4.41), CDK5R1 (*P*_TWAS YBL_ = 1.22 × 10^− 3^, mRNA FC = 2.24), MMP24 (*P*_TWAS MS_ = 1.49 × 10^− 3^, mRNA FC = 2.41), and DDX20 (*P*_TWAS YBL_ = 1.79 × 10^− 3^, mRNA FC = 2.69).

**Table 2:** The top 10 candidate genes detected by TWAS and mRNA expression profile analysis for knee OA, CT, and SDH

Chondropathies	Genes	TWAS		mRNA expression	
		Tissue	*P*_TWAS_	FC	*P*_mRNA_
Knee OA	NSA2	MS/YBL	2.47 × 10^−4^/ 3.30 × 10^− 4^	− 2.16	2.12 × 10^− 2^
	CSF1R	YBL	3.95 × 10^−4^	3.47	2.83 × 10^−2^
	MSC	YBL	9.51 × 10^−4^	4.41	2.03 × 10^−2^
	CDK5R1	YBL	1.22 × 10^−3^	2.24	4.71 × 10^−2^
	MMP24	MS	1.49 × 10^−3^	2.41	2.64 × 10^−2^
	ARMC1	MS/ YBL	1.62 × 10^−3^/ 8.76 × 10^− 3^	− 2.64	4.43 × 10^− 2^
	NCK1	YBL	1.75 × 10^−3^	− 2.26	4.73 × 10^−2^
	NME6	MS/YBL	9.93 × 10^−3^/ 1.75 × 10^− 3^	− 2.93	2.22 × 10^− 3^
	DDX20	YBL	1.79 × 10^−3^	2.69	1.15 × 10^−2^
	SCARB1	YBL	2.55 × 10^−3^	− 3.27	6.75 × 10^−3^
CT	SRGN	YBL	1.46 × 10^−3^	3.36	4.18 × 10^−2^
	ETS1	YBL	3.07 × 10^−3^	− 2.16	4.05 × 10^−5^
	PFKFB3	YBL	4.28 × 10^−3^	3.66	7.00 × 10^−3^
	CHURC1	MS/ YBL	1.59 × 10^−2^/ 1.63 × 10^− 2^	− 2.01	1.36 × 10^− 2^
	TRMT112	YBL	1.94 × 10^−2^	− 2.56	1.01 × 10^−6^
	PLEKHA1	MS	2.29 × 10^−2^	− 2.62	7.66 × 10^−5^
	AFF3	YBL	2.62 × 10^−2^	− 2.35	2.08 × 10^−4^
	S100P	YBL	3.31 × 10^−2^	5.49	2.29 × 10^−2^
	PSMC5	YBL	4.68 × 10^−2^	− 2.22	4.21 × 10^−7^
	C1QTNF4	MS	4.96 × 10^−2^	3.32	2.38 × 10^−5^
SDH	SUPV3L1	YBL	3.59 × 10^−3^	3.22	–
	ZNF195	YBL	6.67 × 10^− 3^	2.25	–
	HBG2	YBL	2.36 × 10^−2^	3.91	–
	VAMP4	YBL	4.77 × 10^−2^	2.23	–

Note: MS denotes muscle skeleton. YBL denotes peripheral blood. FC denotes mRNA expression fold change. TWAS means transcriptome-wide association. *P*_TWAS_ means the *P* value of TWAS. *P*_mRNA_ means the *P* value of mRNA expression. The *P*_mRNA_ of the mRNA expression profile of SDH was not reported in the published study

For CT, 10 overlapped genes were shared by TWAS and mRNA expression profiling (Additional file 6: Table S6), such as SRGN (*P*_TWAS YBL_ = 1.46 × 10^− 3^, mRNA FC = 3.36), ETS1 (*P*_TWAS YBL_ = 3.07 × 10^− 3^, mRNA FC = − 2.16), PFKFB3 (*P*_TWAS YBL_ = 4.28 × 10^− 3^, mRNA FC = 3.66), CHURC1 (*P*_TWAS MS_ = 1.59 × 10^− 2^, *P*_TWAS YBL_ = 1.62 × 10^− 2^, mRNA FC = − 2.01), and S100P (*P*_TWAS YBL_ = 3.31 × 10^− 2^, mRNA FC = 5.49).

For SDH, integrating TWAS and mRNA expression profiles detected four common genes (Additional file 7: Table S7), including SUPV3L1 (*P*_TWAS YBL_ = 3.59 × 10^− 3^, mRNA FC = 3.22), ZNF195 (*P*_TWAS YBL_ = 6.67 × 10^− 3^, mRNA FC = 2.25), HBG2 (*P*_TWAS YBL_ = 2.36 × 10^− 2^, mRNA FC = 3.91), and VAMP4 (*P*_TWAS YBL_ = 4.77 × 10^− 2^, mRNA FC = 2.23).

Interestingly, 4 common genes were found both in knee OA and CT, such as ETS1 (*P*_TWAS YBL_ = 3.07 × 10^− 3^, mRNA FC_OA_ = 2.34, mRNA FC_CT_ = − 2.16), PFKFB3 (*P*_TWAS YBL_ = 4.28 × 10^− 3^, mRNA FC_OA_ = − 4.46, mRNA FC_CT_ = 3.66), CHURC1 (*P*_TWAS MS_ = 1.59 × 10^− 2^, *P*_TWAS YBL_ = 1.63 × 10^− 2^, mRNA FC_OA_ = − 5.32, mRNA FC_CT_ = − 2.01), and C1QTNF4 (*P*_TWAS MS_ = 4.96 × 10^− 2^, mRNA FC_OA_ = − 4.82, mRNA FC_CT_ = 3.32).

### Gene ontology (GO) enrichment analyses

The identified common genes shared by TWAS and mRNA expression profiles were subjected to GO enrichment analysis. For knee OA, we identified 73 GO terms (Table 3, Additional file 8: Table S8), such as mitochondrial protein complex (*P* = 7.31 × 10^− 5^), mitochondrial membrane part (*P* = 1.81 × 10^− 4^), oxidoreductase complex (*P* = 4.87 × 10^− 4^), and regulation of lipid catabolic process (*P* = 4.40 × 10^− 4^). For CT, 3 GO terms were detected, including response to tumor necrosis factor (*P* = 9.22 × 10^− 5^), response to cytokine (*P* = 1.13 × 10^− 4^), and positive regulation of defense response (*P* = 3.46 × 10^− 4^). The transition metal ion binding (*P* = 3.55 × 10^− 4^) was the only GO term detected for SDH.

**Table 3:** The top 10 gene ontology terms identified for knee OA

Gene ontology	Tissue	*P* value	Adjusted *P* value
GO_MATURATION_OF_LSU_RRNA	MS	1.18 × 10^− 6^	5.21 × 10^− 3^
GO_NADH_DEHYDROGENASE_COMPLEX	MS	4.56 × 10^− 5^	1.84 × 10^− 2^
GO_MITOCHONDRIAL_PROTEIN_COMPLEX	MS	7.31 × 10^− 5^	1.84 × 10^− 2^
GO_MITOCHONDRIAL_MEMBRANE_PART	MS	1.81 × 10^− 4^	2.10 × 10^− 2^
GO_RESPIRATORY_CHAIN	MS	2.90 × 10^− 4^	2.81 × 10^− 2^
GO_OXIDOREDUCTASE_COMPLEX	MS	4.87 × 10^− 4^	3.18 × 10^− 2^
GO_INNER_MITOCHONDRIAL_MEMBRANE_PROTEIN_COMPLEX	MS	7.14 × 10^− 4^	3.45 × 10^− 2^
GO_PURINE_NUCLEOBASE_BIOSYNTHETIC_PROCESS	YBL	4.75 × 10^− 6^	2.11 × 10^− 2^
GO_NUCLEOBASE_BIOSYNTHETIC_PROCESS	YBL	1.74 × 10^− 5^	3.86 × 10^− 2^
GO_PURINE_NUCLEOBASE_METABOLIC_PROCESS	YBL	2.82 × 10^− 5^	4.17 × 10^− 2^

Note: MS denotes muscle skeleton. YBL denotes peripheral blood

### IHC validation

**Fig. 1:**
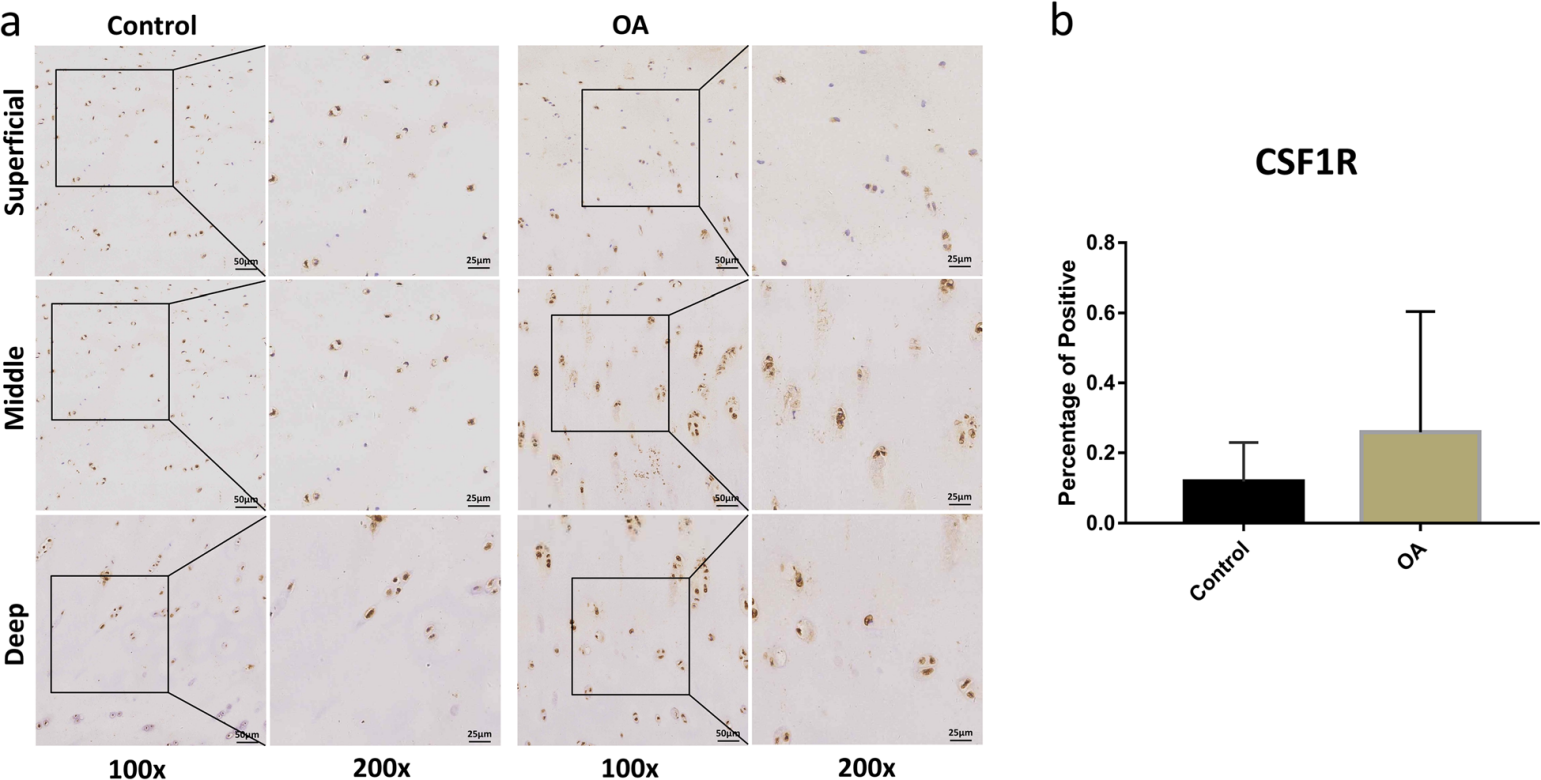
The protein expression levels of CSF1R in the knee OA and control cartilage. Immunostaining for CSF1R (**a**) in the superficial, middle, and deep layers of OA and control cartilage. Scale bar stands for 50 μm in × 100 pictures and 25 μm in × 200 pictures. The expression of CSF1R protein in the OA chondrocytes (0.25%) was higher than in the normal chondrocytes (0.11%), but with no statistical significance (*P* = 0.5304) (**b**). ImageJ was used to analyze the percentage of positive chondrocytes

**Fig. 2:**
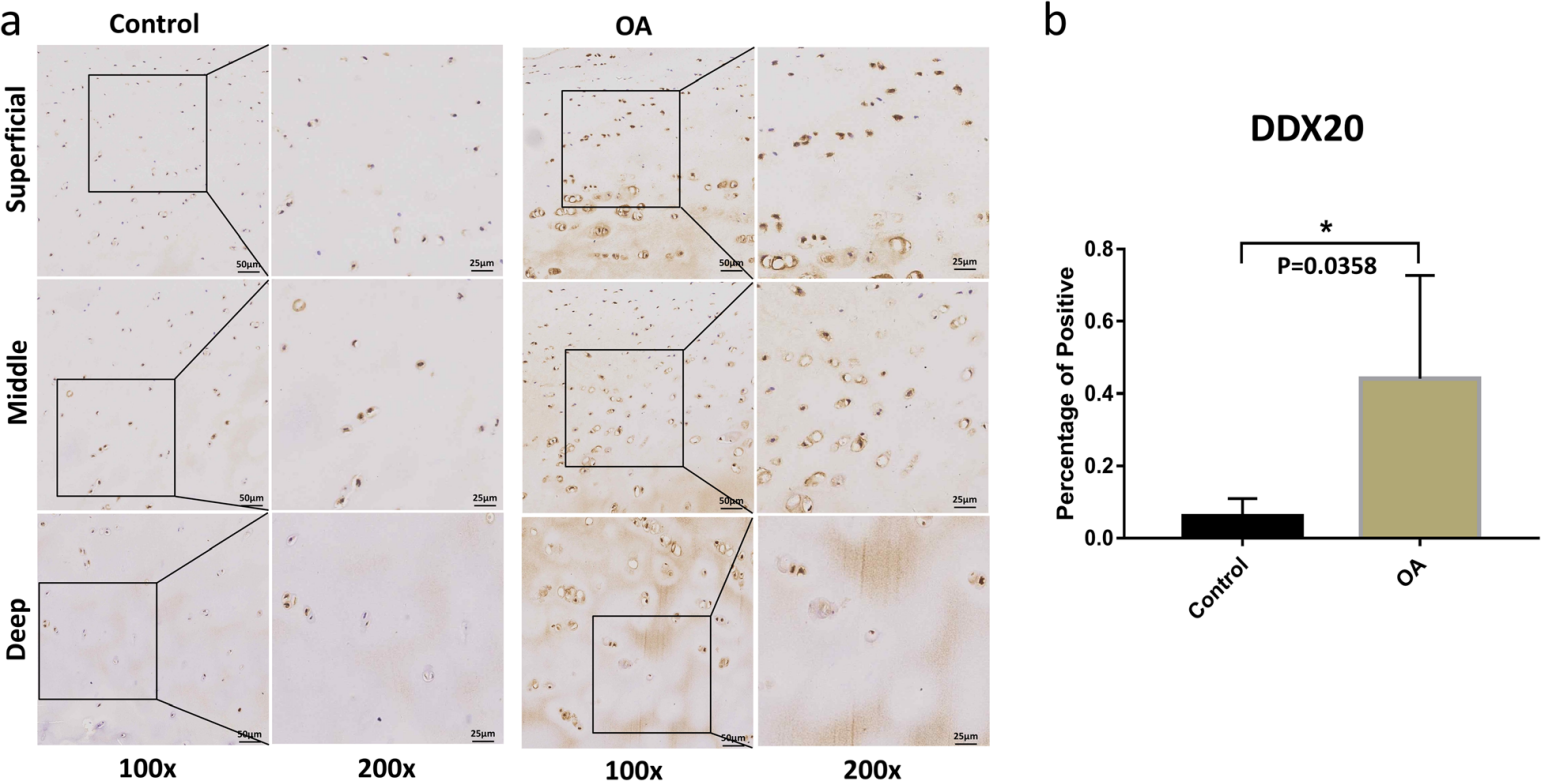
The protein expression levels of DDX20 in the knee OA and control cartilage. Immunostaining for DDX20 (**a**) in the superficial, middle, and deep layers of OA and control cartilage. Scale bar stands for 50 μm in × 100 pictures and 25 μm in × 200 pictures. The expression of DDX20 protein was significantly overexpressed (*P = 0.0358) in OA chondrocytes (0.44%) compared to that in healthy cartilage (0.06%) (**b**). ImageJ was used to analyze the percentage of positive chondrocytes

The expression levels of CSF1R and DDX20 were evaluated in the cartilage specimens from knee OA patients and healthy controls (Figs. 1 and 2). IHC results showed that the percentage of positive chondrocytes of DDX20 in knee OA cartilage (0.44%) was significantly higher compared to heathy cartilage (0.06%, *P* = 0.0358). CSF1R also showed overexpression in knee OA cartilage compared to heathy controls (0.25% vs. 0.11%), but not statistically significant (*P* = 0.5304).

## Discussion

Recently, GWAS have successfully identified amount of candidate variants for complex diseases. However, the identified loci are almost located at non-coding regions and could hardly explain the relative risk [12]. Therefore, in order to yield an insight on the mechanism of chondropathies, we performed TWAS by using large-scale GWAS data of chondropathies. Then, the results of TWAS were integrated with different mRNA expression data of three types of chondropathies to find causal genes for knee OA, CT, and SDH.

Integrative TWAS and mRNA expression profiles detected multiple candidate genes for knee OA, such as DDX20 and NSA2. DDX20, a member of the Asp-Glu-Ala-Asp (DEAD) box protein family, a component of microRNA-containing ribonucleoprotein complexes, encodes an RNA helicase. Previous study suggested that miRNA-140 plays a central role in DDX20 deficiency-related pathogenesis, taking part in the classical nuclear factor-κB (NF-κB) pathway and targeting DNA metyltransferase 1 expression [21]. Recently, the NF-κB pathway has been found to regulate chondrocyte proliferation and differentiation and maintain endochondral ossification [22]. NSA2 was the most significant gene identified by TWAS for chondropathies, also detected by the mRNA expression profiling of knee OA cartilage. NSA2 encodes Nop seven-associated 2 ribosome biogenesis factor. It is a nucleolar protein required for ribosome biogenesis and involved in cell cycle regulation and proliferation [23]. Recently, NSA2 has been reported as the candidate gene for diabetic nephropathy and is related to the TGF-β1 pathway [24].

Besides, we have identified several significant genes associated with CT, such as SRGN. Its official full name is serglycin. The protein of this gene is best known as a hematopoietic cell granule proteoglycan, which may be vital for neutralizing hydrolytic enzymes and involved in the granule-mediated apoptosis. SRGN is over expressed in tumor cells and associated with tumor cell aggressiveness and poor prognosis in cancers [25]. Another study detected SRGN is markedly synthesized by cancer and stromal cells in malignant cancers [26], indicating a critical role of SRGN in the procession of cancer.

Another notable gene for CT is S100P, which is a member of the S100 family of proteins containing 2 EF-hand calcium-binding motifs. The protein encoded by this gene could not only bind Ca2+, Zn2+, and Mg2+, but also play an important role in the progress of various tumors [27]. Yang et al. have illustrated the relationship between blood-based S100P methylation and breast cancer [28]. Moreover, Piltti et al. demonstrated that S100P was the only significantly changed S100 protein family member and its expression was 2.5 times higher than the control in human chondrosarcoma HCS-2/8 cells [29]. This result is almost coincident with our findings about S100P, showing that S100P was overexpressed in CT and may be a causal gene for the disease.

Interestingly, we also found 4 common genes both in knee OA and CT, such as PFKFB3. It belongs to a family of bifunctional proteins involved in both the synthesis and degradation of fructose-2,6-bisphosphate, and in charge of glycolysis in eukaryotes. A previous study has demonstrated the important role of PFKFB3 in regulating the glycolytic metabolism in human OA cartilage [30]. Besides, PFKFB3 is also identified in glycolysis, cell proliferation, and tumor growth in the procession of tumor and inflammation [31]. Taken together, it would be of significant importance to further detect the role of PFKFB3 involved in the development of knee OA and CT, and the potential treatment target of PFKFB3 for both of them.

For SDH, we detected 4 causal genes. For instance, ZNF195, zinc finger protein 195, located at chromosome 11p15.5, is a member of the Krueppel C2H2-type zinc-finger protein family. The family mainly includes transcription factors and participants in various cellular processes. Previous studies have shown that ZNF195 is related to various cancers, such as cutaneous T cell lymphoma [32]. The expression of ZNF195 was closely related to gemcitabine sensitivity in head and neck squamous cell carcinoma (HNSCC) and decreased in the low level of oxygen in first trimester human trophoblast cells [33]. However, few researches have demonstrated the function of ZNF195, and none about this gene in SDH. Further study is needed to identify the mechanism of ZNF195 involved in the development and progression of SDH.

Multiple GO terms were enriched in the candidate genes of the three types of chondropathies. For knee OA, mitochondria were identified as the major parts of GO cellular component terms, such as mitochondrial membrane part (GO:0044455), inner mitochondrial membrane protein complex (GO:009800), and mitochondrial protein complex (GO:0098798). It has been identified that mitochondria are important regulators of cellular function and survival and play a vital role in aging-related diseases, especially in OA [34]. Our results further confirmed the importance of mitochondria and highlighted the mitochondrial membrane in the etiology of knee OA. In addition, the association of oxidoreductase (GO:1990204, GO:0034614, GO:0055114, GO:0016491) and lipid (GO:0050994, GO:0046890, GO:0046889) GO terms was found as the main biological process and molecular function terms changed in knee OA. Poloma et al. [35] have demonstrated that decreasing mitochondrial membrane potential is related to increasing reactive oxygen species (ROS) production and cell death in OA. Tang et al. [36] indicated that reversing oxidative stress-mediated mitochondrial membrane potential collapse could be an effective way for OA protection against mitochondrial dysfunction. On the other hand, obesity is a major risk factor for OA. The dyslipidemia plays an important role in obesity-induced OA [37]. Lipid deposition in the joint was detected at the early stages of OA before histological changes [38]. Multiple lipid metabolism-related genes have been identified dramatically reduced in OA articular cartilage compared to normal samples, such as ATP-binding-cassette transporter A1 (ABCA1), apoliproprotein A1 (ApoA1), liver X receptor alpha (LXR α), and liver X receptor beta (LXR β) [39]. Therefore, the function of mitochondria, oxidoreductase, and lipid could not be underestimated in the OA development.

For CT, we detected that the biological processes of CT are mainly related to tumor necrosis factor (GO:0034612), cytokine (GO:0034097), and positive regulation of defense response (GO:0031349). Many studies have shown that TNF as well as cytokine, such as IL-1, IL-6, and IL-17, has been involved in the cartilage degradation [40]. It has been identified that TNF regulates the inflammatory cascade, and IL-1β is related to cartilage destruction. TNF and IL-1β play a critical role in the cartilage degradation of OA [41]. The findings in our study may give a novel molecular mechanism insight in CT procession.

For SDH, only one GO molecular function term was significantly enriched, which is transition metal ion binding (GO:0046914). There are eight metals involved in the biologically relevant transition metals, including vanadium, manganese, iron, copper, cobalt, nickel, molybdenum, and silver. Copper deficiency has been identified as a critical factor in antioxidant defenses, mitochondrial energy production, and in iron metabolism in blood and muscles in myeloneuropathy [42]. Therefore, it would be worthy to deeply evaluate the other transition metals in SDH procession.

There are two innovations in our study. First, given the common biological processes shared by different types of chondropathies, we conducted a TWAS using chondropathies as phenotypes. This approach may help us to detect novel susceptibility genes contributing to the overlapped genetic mechanism of various chondropathies. Another innovation is that we integrated TWAS results with different mRNA expression profiles of three disorders of chondropathies to find the common genes. The results of these common genes could be more reliable because they are not only based on the TWAS results, but also verify the TWAS results according to different mRNA profiles. Additionally, the aim of this study is to genome-wide scan candidate genes for chondropathies, through integrating TWAS and mRNA expression profile data. To validate the accuracy of integrative analysis results, two knee OA associated candidate genes were selected for IHC validation. Further functional studies are needed to confirm our findings and clarify the potential mechanism of identified candidate genes involved in the development of chondropathies.

## Conclusions

In conclusion, we conducted an integrative analysis of TWAS and mRNA expression profiles and identified multiple knee OA-, CT-, and SDH-related candidate genes and GO terms. Our findings may provide a novel insight into the pathogenesis of chondropathies.

## Additional files

Additional file 1: **Table S1.** The characteristic of the mRNA expression study samples. (DOCX 14 kb)

Additional file 2: **TableS2.** The basic characteristics of study samples for IHC. (DOCX 13 kb)

Additional file 3: **Table S3.** TWAS identified significant genes in MS for chondropathies. (DOCX 29 kb)

Additional file 4: **Table S4.** TWAS identified significant genes in YBL for chondropathies. (DOCX 34 kb)

Additional file 5: **Table S5.** Interative analyses identified candidate genes for knee OA. (DOCX 26 kb)

Additional file 6: **Table S6.** Interative analyses identified causal genes for cartilage tumor. (DOCX 16 kb)

Additional file 7: **Table S7.** Interative analyses identified causal genes for spinal disc herniation. (DOCX 15 kb)

Additional file 8: **Table S8.** GO enrichment analyses results for knee OA. (DOCX 23 kb)

## Data Availability

The datasets used and/or analyzed during the current study are available from the corresponding author on reasonable request.

## Abbreviations

ABCA1: ATP-binding-cassette transporter A1
ApoA1: Apoliproprotein A1
CT: Cartilage tumor
FC: Fold change
FUMA: Functional mapping and annotation
GO: Gene ontology
GWAS: Genome-wide association studies
HNSCC: Head and neck squamous cell carcinoma
HRP: Horseradish peroxidase
IL-1β: Interleukin 1β
IVD: Intervertebral disc
LIMMA: Linear models for microarray
LXR α: Liver X receptor alpha
LXR β: Liver X receptor beta
MS: Muscle skeleton
NF-κB: Nuclear factor-κB
OA: Osteoarthritis
qRT-PCR: Quantitative reverse transcription polymerase chain reaction
ROS: Reactive oxygen species
RT: Room temperature
SDH: Spinal disc herniation
TNF-α: Tumor necrosis factor α
TWAS: Transcriptome-wide association study
YBL: Peripheral blood
