# Supplementary material for: Integrating transcriptome-wide study and mRNA expression profiles yields novel insights into the biological mechanism of chondropathies
Source: Arthritis Res Ther. 2019 Aug 27;21:194. doi: 10.1186/s13075-019-1978-8 (PMC6712880; doi:10.1186/s13075-019-1978-8)
Supplement: Supplementary file 1 — Table S1. The characteristic of the mRNA expression study samples. (DOCX 14 kb) [file 13075_2019_1978_MOESM1_ESM.docx]

**Table S1**. The characteristic of the mRNA expression study samples

| **Groups** | | | **Size** | |  | **Age (years)** | |  | **Male/Female** | | **Microarray chip** |
| --- | --- | --- | --- | --- | --- | --- | --- | --- | --- | --- | --- |
|  | | **Case** | **Control** |  | **Case** | **Control** |  | **Case** | **Control** |  |  |
| Knee OA | | 6 | | 5 |  | 57-77 | 10-17 |  | 1/5 | 3/2 | Arraystar Human lncRNA Array v2.0 |
| CT | | 7 | | 6 |  | 15-39 | - |  | 3/4 | - | Illumina BeadArray v3.0 |
| SDH | | 10 | | 10 |  | 21-43 | 28-55 |  | 4/6 | 5/5 | Agilent’s Human 1A |
