# Supplementary material for: Integrating transcriptome-wide study and mRNA expression profiles yields novel insights into the biological mechanism of chondropathies
Source: Arthritis Res Ther. 2019 Aug 27;21:194. doi: 10.1186/s13075-019-1978-8 (PMC6712880; doi:10.1186/s13075-019-1978-8)
Supplement: Supplementary file 2 — TableS2. The basic characteristics of study samples for IHC. (DOCX 13 kb) [file 13075_2019_1978_MOESM2_ESM.docx]

**TableS2.** The basic characteristics of study samples for IHC

|  | Heathy control | Knee OA |
| --- | --- | --- |
| N | 4 | 5 |
| Age (Mean±SD) | 49.7±10.97 | 64.6±5.68 |
| Male/Female | 3/1 | 3/2 |

Note: Independent *t* test showed that the two groups had no significant difference (*P*=0.128) in ages.
