# Supplementary material for: Integrating transcriptome-wide study and mRNA expression profiles yields novel insights into the biological mechanism of chondropathies
Source: Arthritis Res Ther. 2019 Aug 27;21:194. doi: 10.1186/s13075-019-1978-8 (PMC6712880; doi:10.1186/s13075-019-1978-8)
Supplement: Supplementary file 3 — Table S3. TWAS identified significant genes in MS for chondropathies. (DOCX 29 kb) [file 13075_2019_1978_MOESM3_ESM.docx]

**Table S3 TWAS identified significant genes in MS for chondropathies**

| Gene | CHR | GWAS SNP | GWAS Z | EQTL SNP | EQTL Z | TWAS Z | TWAS P |
| --- | --- | --- | --- | --- | --- | --- | --- |
| NSA2 | 5 | rs1048167 | 3.69 | rs6877188 | 14.78 | 3.67 | 2.47E-04 |
| TP53I13 | 17 | rs1017529 | 3.47 | rs3110496 | -3.93 | 3.65 | 2.63E-04 |
| FPGT | 1 | rs1601153 | -3.89 | rs11210395 | -7.4 | 3.56 | 3.69E-04 |
| PLOD2 | 3 | rs4681298 | 3.24 | rs3762690 | 6.19 | 3.52 | 4.34E-04 |
| RSRC1 | 3 | rs7624303 | -3.77 | rs11708784 | -4.87 | 3.51 | 4.54E-04 |
| MTFR1L | 1 | rs1835308 | -3.46 | rs213624 | -10.64 | 3.48 | 5.00E-04 |
| RP1-317E23.7 | 1 | rs1835308 | -3.46 | rs213638 | -9.54 | 3.46 | 5.48E-04 |
| RP11-538P18.2 | 3 | rs7624303 | -3.77 | rs11708784 | -5.68 | 3.42 | 6.32E-04 |
| RP11-731C17.2 | 3 | rs6805715 | 3.94 | rs6439663 | -6.93 | 3.23 | 1.22E-03 |
| MMP24 | 20 | rs932562 | -2.81 | rs6060341 | -5.76 | 3.18 | 1.49E-03 |
| ARMC1 | 8 | rs4327888 | -3.65 | rs4737740 | -4.71 | -3.15 | 1.62E-03 |
| TNNI3 | 19 | rs891187 | 3.55 | rs11671293 | 5.81 | 3.12 | 1.80E-03 |
| PSMD5 | 9 | rs10985016 | -3.57 | rs1060817 | 10.85 | -3.1 | 1.94E-03 |
| RP1-93H18.7 | 6 | rs7770769 | 3.17 | rs9320557 | 3.51 | 3.09 | 2.02E-03 |
| IFT88 | 13 | rs4770118 | 2.89 | rs6490598 | 5.53 | -3.02 | 2.56E-03 |
| RP5-966M1.6 | 3 | rs4687657 | -3.02 | rs13083728 | 7.05 | -2.96 | 3.07E-03 |
| CTC-228N24.3 | 5 | rs6892624 | -3.6 | rs3749748 | -8.51 | -2.96 | 3.08E-03 |
| RP11-408A13.4 | 9 | rs2382492 | 3.15 | rs2382492 | 5.52 | 2.95 | 3.16E-03 |
| RP11-344N10.5 | 10 | rs7899274 | 3.16 | rs7908682 | 4.12 | 2.94 | 3.29E-03 |
| TUSC1 | 9 | rs4978005 | 3.15 | rs12348 | 5.49 | 2.92 | 3.55E-03 |
| TYMS | 18 | rs505140 | 2.8 | rs699517 | -5.47 | 2.9 | 3.68E-03 |
| MED4-AS1 | 13 | rs9567923 | 2.58 | rs7996581 | -5.4 | -2.86 | 4.27E-03 |
| AC016747.3 | 2 | rs7599341 | -2.8 | rs1177274 | 4.69 | 2.83 | 4.72E-03 |
| PTGR1 | 9 | rs1475106 | 3.3 | rs10759502 | -5.74 | -2.81 | 4.96E-03 |
| FAM149B1 | 10 | rs16930753 | 3.27 | rs4529828 | 6.74 | 2.81 | 5.03E-03 |
| GOLGA8B | 15 | rs11630666 | 3.64 | rs8041207 | -5.06 | 2.79 | 5.27E-03 |
| MTFR1 | 8 | rs4327888 | -3.65 | rs7008671 | 8.22 | -2.79 | 5.32E-03 |
| ITIH4-AS1 | 3 | rs4687657 | -3.02 | rs4687657 | 5.48 | -2.78 | 5.39E-03 |
| ATP5C1 | 10 | rs1980856 | 4.07 | rs2057604 | -7.05 | 2.76 | 5.85E-03 |
| AC009404.2 | 2 | rs11690896 | -3.5 | rs17499330 | -4.2 | 2.74 | 6.22E-03 |
| RP11-274B21.2 | 7 | rs10225988 | -2.77 | rs2402923 | 9.83 | -2.72 | 6.53E-03 |
| RP11-274B21.4 | 7 | rs10225988 | -2.77 | rs6467202 | 10.76 | -2.72 | 6.54E-03 |
| ZNF571-AS1 | 19 | rs1148395 | -2.88 | rs11666497 | 4.09 | 2.72 | 6.55E-03 |
| CTD-2203K17.1 | 5 | rs4866357 | 3.24 | rs3756663 | -7.26 | 2.71 | 6.63E-03 |
| L3HYPDH | 14 | rs893516 | -3.48 | rs8660 | -9.9 | 2.7 | 7.02E-03 |
| DHFR | 5 | rs6859084 | -3.39 | rs13161245 | -12.42 | -2.69 | 7.11E-03 |
| BCL7C | 16 | rs34453065 | -3.58 | rs17839549 | 6.55 | -2.69 | 7.13E-03 |
| EIF6 | 20 | rs932562 | -2.81 | rs2425044 | 9.9 | -2.68 | 7.47E-03 |
| NDFIP1 | 5 | rs17097801 | 3.41 | rs12653848 | -7.08 | 2.67 | 7.48E-03 |
| RP11-274B21.3 | 7 | rs10225988 | -2.77 | rs2402923 | 9.51 | -2.67 | 7.48E-03 |
| PHLPP2 | 16 | rs16973286 | 3.08 | rs7193549 | 6.02 | 2.67 | 7.58E-03 |
| POLR2J3 | 7 | rs12534337 | -2.69 | rs4729791 | 6.78 | -2.67 | 7.66E-03 |
| RP11-305E17.6 | 1 | rs2149194 | -2.75 | rs2149194 | 3.93 | -2.67 | 7.66E-03 |
| RP4-614O4.11 | 20 | rs932562 | -2.81 | rs2425044 | 9.19 | -2.64 | 8.37E-03 |
| WDR11 | 10 | rs2420931 | -3.22 | rs10886788 | -6.85 | 2.63 | 8.49E-03 |
| AKAP11 | 13 | rs12584909 | -3.2 | rs9594733 | 6.53 | -2.63 | 8.54E-03 |
| PCMTD2 | 20 | rs7264220 | -3.48 | rs11905831 | -5.42 | 2.61 | 9.06E-03 |
| PIGCP1 | 11 | rs3802789 | -2.58 | rs12276196 | 7.04 | -2.6 | 9.25E-03 |
| RWDD1 | 6 | rs7770769 | 3.17 | rs2250263 | -4.72 | -2.6 | 9.32E-03 |
| TCP11L1 | 11 | rs3802789 | -2.58 | rs7926030 | 8.91 | -2.6 | 9.42E-03 |
| NUDT13 | 10 | rs16930753 | 3.27 | rs12569470 | -4.55 | -2.6 | 9.46E-03 |
| CDC25A | 3 | rs3895736 | 2.85 | rs13059037 | 9.64 | -2.59 | 9.50E-03 |
| MOB3C | 1 | rs522025 | -2.65 | rs11211319 | -4.44 | 2.59 | 9.54E-03 |
| FNDC5 | 1 | rs476153 | -3.06 | rs785275 | 7.92 | -2.58 | 9.88E-03 |
| NME6 | 3 | rs3895736 | 2.85 | rs9819094 | -7.09 | 2.58 | 9.93E-03 |
| NCKIPSD | 3 | rs3895736 | 2.85 | rs12493578 | -7.79 | -2.58 | 9.98E-03 |
| MAPRE3 | 2 | rs7564363 | -3.03 | rs11126806 | 5.14 | -2.58 | 1.00E-02 |
| RP11-27I1.4 | 9 | rs10985016 | -3.57 | rs10985148 | 4.95 | -2.57 | 1.03E-02 |
| ABCA11P | 4 | rs4130382 | 3.19 | rs17721347 | -3.76 | -2.56 | 1.06E-02 |
| TRPT1 | 11 | rs1783811 | -2.65 | rs11603192 | 9.81 | 2.55 | 1.06E-02 |
| RP11-157J24.2 | 6 | rs9502833 | -2.75 | rs4367416 | 9.35 | -2.53 | 1.14E-02 |
| BLMH | 17 | rs12947084 | 3.64 | rs35154099 | -3.39 | -2.52 | 1.16E-02 |
| RP11-274B21.1 | 7 | rs10225988 | -2.77 | rs2402923 | 9.8 | -2.51 | 1.21E-02 |
| CEP76 | 18 | rs2847254 | 3.53 | rs12455481 | 5.18 | -2.5 | 1.25E-02 |
| ACCS | 11 | rs7111879 | 2.92 | rs7951555 | 9.11 | -2.5 | 1.26E-02 |
| C15orf40 | 15 | rs4842993 | 3.46 | rs1568657 | 5.67 | 2.49 | 1.26E-02 |
| PSMD5-AS1 | 9 | rs10985016 | -3.57 | rs4837796 | 14.32 | -2.49 | 1.26E-02 |
| GPR108 | 19 | rs385791 | 3.37 | rs340141 | -8.73 | -2.48 | 1.30E-02 |
| MMP24-AS1 | 20 | rs932562 | -2.81 | rs6060341 | -4.78 | 2.48 | 1.32E-02 |
| AC103965.1 | 15 | rs12591597 | -2.33 | rs11633534 | -7.75 | -2.47 | 1.37E-02 |
| CLYBL | 13 | rs9300575 | -3 | rs1107450 | -5.59 | -2.47 | 1.37E-02 |
| SH3GLB2 | 9 | rs10113912 | -2.58 | rs17455517 | -6.11 | 2.44 | 1.46E-02 |
| RP11-122G18.5 | 1 | rs3934593 | 2.76 | rs16832908 | -7.99 | -2.44 | 1.48E-02 |
| RP11-212P7.2 | 7 | rs10225988 | -2.77 | rs11973846 | -4.5 | 2.44 | 1.48E-02 |
| RP1-199J3.5 | 6 | rs576231 | 3.39 | rs994198 | -8.35 | -2.44 | 1.49E-02 |
| RAP1A | 1 | rs197394 | 3.2 | rs11803207 | -3.79 | -2.43 | 1.53E-02 |
| CTDP1 | 18 | rs939233 | 3.07 | rs649867 | 4.02 | -2.42 | 1.55E-02 |
| NDUFA10 | 2 | rs13393956 | 2.81 | rs8369 | -6.28 | 2.42 | 1.56E-02 |
| KHK | 2 | rs7564363 | -3.03 | rs12714092 | 9.1 | -2.42 | 1.57E-02 |
| CHURC1 | 14 | rs12884320 | 3.31 | rs2296327 | -8.24 | -2.41 | 1.59E-02 |
| RP11-458F8.2 | 7 | rs4718428 | 2.33 | rs868961 | -4.03 | -2.4 | 1.62E-02 |
| AC018638.1 | 7 | rs10225988 | -2.77 | rs6467202 | 6.4 | -2.4 | 1.66E-02 |
| HMOX2 | 16 | rs11862083 | 2.78 | rs10500325 | 7.21 | 2.39 | 1.67E-02 |
| PHYHD1 | 9 | rs10113912 | -2.58 | rs12553820 | -6.11 | 2.39 | 1.68E-02 |
| USP19 | 3 | rs3895736 | 2.85 | rs2286652 | -3.45 | -2.37 | 1.76E-02 |
| ZNF605 | 12 | rs11147102 | -3.27 | rs11833667 | 4.44 | -2.37 | 1.77E-02 |
| AUH | 9 | rs6479344 | 2.75 | rs296646 | 3.84 | 2.37 | 1.80E-02 |
| ANKRD44 | 2 | rs4850776 | 2.61 | rs11885072 | 5 | -2.36 | 1.83E-02 |
| TMEM110 | 3 | rs4687657 | -3.02 | rs2276816 | 7.09 | -2.36 | 1.83E-02 |
| NUDT2 | 9 | rs1571401 | -3.47 | rs10972063 | 13.3 | 2.36 | 1.85E-02 |
| RP11-85F14.5 | 3 | rs6805715 | 3.94 | rs1805300 | -5.63 | 2.36 | 1.85E-02 |
| RP11-561C5.4 | 15 | rs8187736 | 2.58 | rs9744503 | 4.53 | 2.35 | 1.87E-02 |
| NCLN | 19 | rs3746124 | 2.83 | rs311621 | -8.52 | -2.34 | 1.94E-02 |
| PSMA6 | 14 | rs10140560 | 3.83 | rs4982250 | 7.05 | -2.33 | 1.96E-02 |
| RP11-514P8.8 | 7 | rs12534337 | -2.69 | rs2229796 | 6.12 | -2.33 | 1.96E-02 |
| RASA4B | 7 | rs12534337 | -2.69 | rs2229796 | 5.89 | -2.33 | 1.98E-02 |
| GNAL | 18 | rs9948889 | 2.9 | rs2903236 | 4.55 | 2.31 | 2.08E-02 |
| PDCL3P4 | 3 | rs7629753 | -2.58 | rs7651721 | 6.07 | -2.29 | 2.18E-02 |
| LINC00926 | 15 | rs12440670 | 2.85 | rs2544129 | 7.03 | -2.29 | 2.21E-02 |
| IMPACT | 18 | rs4800536 | 2.75 | rs550010 | -3.78 | 2.28 | 2.27E-02 |
| PLEKHA1 | 10 | rs3763763 | 2.99 | rs4751890 | 4.55 | 2.27 | 2.29E-02 |
| ANKRD10 | 13 | rs2391890 | 3.01 | rs2893386 | 4.17 | -2.26 | 2.37E-02 |
| CTD-2561B21.7 | 17 | rs9319606 | -2.58 | rs6565507 | 4.71 | -2.26 | 2.38E-02 |
| ECD | 10 | rs16930753 | 3.27 | rs12569470 | -3.73 | -2.26 | 2.38E-02 |
| ABHD8 | 19 | rs11879994 | 3.86 | rs11086067 | -5.92 | -2.25 | 2.41E-02 |
| ATP6V1E2 | 2 | rs9808496 | -4.2 | rs1868844 | 7.92 | -2.25 | 2.42E-02 |
| GALNT16 | 14 | rs11844404 | 3.3 | rs7140358 | -5.13 | -2.25 | 2.42E-02 |
| RP11-386G11.5 | 12 | rs10783282 | 2.58 | rs10783299 | -6.06 | 2.25 | 2.43E-02 |
| MFF | 2 | rs1134745 | 2.9 | rs1134745 | -5.65 | -2.25 | 2.44E-02 |
| CDPF1 | 22 | rs3788727 | -2.6 | rs16995067 | -4.66 | -2.25 | 2.47E-02 |
| GLTP | 12 | rs11068103 | -2.81 | rs7966820 | 6.38 | -2.25 | 2.47E-02 |
| SRRD | 22 | rs596633 | 3.02 | rs9613102 | -3.85 | -2.24 | 2.48E-02 |
| FAM86JP | 3 | rs2138214 | 2.63 | rs1127717 | 10.94 | 2.24 | 2.50E-02 |
| POLI | 18 | rs1403884 | -2.58 | rs3730783 | -7.27 | -2.24 | 2.50E-02 |
| CGREF1 | 2 | rs7564363 | -3.03 | rs12714092 | 5.89 | -2.24 | 2.53E-02 |
| RPL23AP7 | 2 | rs11677881 | 2.58 | rs4849261 | 9.79 | 2.24 | 2.54E-02 |
| SMIM20 | 4 | rs11735002 | 2.94 | rs9647441 | -9.63 | -2.22 | 2.62E-02 |
| NEU3 | 11 | rs529513 | 2.58 | rs594144 | -5.33 | -2.22 | 2.67E-02 |
| C9orf78 | 9 | rs7019254 | 2.73 | rs2274508 | -4.49 | -2.21 | 2.68E-02 |
| PBLD | 10 | rs12570981 | -3.04 | rs34888891 | 6.88 | -2.21 | 2.70E-02 |
| PDCL3 | 2 | rs1437968 | -2.65 | rs13422218 | 6.11 | 2.2 | 2.77E-02 |
| C1orf192 | 1 | rs3934593 | 2.76 | rs16832908 | -5.51 | -2.2 | 2.79E-02 |
| GCNT4 | 5 | rs1048167 | 3.69 | rs3935470 | -3.15 | 2.2 | 2.79E-02 |
| TRIM66 | 11 | rs11602553 | -3.54 | rs10840112 | 8.86 | 2.2 | 2.80E-02 |
| ACOX2 | 3 | rs11708352 | -3.02 | rs13097249 | -3.33 | -2.2 | 2.81E-02 |
| RP11-54C4.1 | 5 | rs11950215 | -3.21 | rs1062177 | -6.81 | -2.2 | 2.81E-02 |
| C15orf41 | 15 | rs12899716 | -2.85 | rs6495850 | 3.49 | -2.19 | 2.83E-02 |
| MTCH2 | 11 | rs4752977 | -2.86 | rs12361256 | 4.78 | 2.19 | 2.84E-02 |
| RASL10B | 17 | rs8065286 | -3.02 | rs9894813 | 4.66 | -2.19 | 2.84E-02 |
| TAF1B | 2 | rs4668664 | 2.61 | rs2245344 | -8.36 | -2.19 | 2.85E-02 |
| RP11-24N18.1 | 16 | rs34763945 | -3.19 | rs2070962 | 3.75 | 2.19 | 2.88E-02 |
| RP11-95D17.1 | 2 | rs4668664 | 2.61 | rs2245344 | -6.62 | -2.18 | 2.89E-02 |
| ELK4 | 1 | rs10900494 | 3.06 | rs7530988 | -4.27 | -2.17 | 2.98E-02 |
| CSTB | 21 | rs2838280 | -3.19 | rs9985006 | -7.94 | -2.17 | 3.00E-02 |
| RP11-95I16.4 | 10 | rs2420931 | -3.22 | rs7079527 | -8.96 | 2.17 | 3.00E-02 |
| CLHC1 | 2 | rs2588512 | 2.69 | rs7349405 | 10.99 | -2.17 | 3.01E-02 |
| MED4 | 13 | rs943238 | -2.67 | rs9526450 | -5.72 | -2.17 | 3.01E-02 |
| ACADM | 1 | rs1146644 | 2.87 | rs11161510 | 8.59 | -2.17 | 3.03E-02 |
| KIAA1033 | 12 | rs11112469 | 3.53 | rs1196852 | -9.14 | -2.16 | 3.06E-02 |
| RP11-119F19.2 | 10 | rs2490275 | -3.77 | rs2341958 | 5.06 | -2.16 | 3.09E-02 |
| AP001877.1 | 11 | rs6483469 | 3.02 | rs11021309 | 4.72 | -2.16 | 3.10E-02 |
| PDHX | 11 | rs286900 | -2.6 | rs17435469 | 6.7 | -2.16 | 3.11E-02 |
| TTC3 | 21 | rs2051399 | -4.07 | rs6517404 | 6.46 | -2.16 | 3.11E-02 |
| PCNXL4 | 14 | rs1957309 | -2.58 | rs219326 | 6.09 | -2.14 | 3.28E-02 |
| USP20 | 9 | rs7019254 | 2.73 | rs3758337 | 5.69 | 2.13 | 3.30E-02 |
| GBA2 | 9 | rs3750434 | 2.33 | rs10758321 | 6.39 | 2.13 | 3.31E-02 |
| FGFRL1 | 4 | rs4130382 | 3.19 | rs34627176 | -5.7 | 2.13 | 3.32E-02 |
| PDCD1LG2 | 9 | rs911735 | 3.07 | rs343471 | 4.09 | 2.13 | 3.35E-02 |
| HAUS4 | 14 | rs7469 | 2.91 | rs11624528 | 5.85 | 2.12 | 3.37E-02 |
| AK3 | 9 | rs159432 | 3.52 | rs387413 | -9.89 | -2.11 | 3.52E-02 |
| TRIP11 | 14 | rs2295168 | 3.4 | rs910369 | 4.33 | -2.1 | 3.55E-02 |
| TUBGCP2 | 10 | rs1573040 | 3.02 | rs11818839 | 5.13 | 2.09 | 3.66E-02 |
| CTD-2538C1.2 | 19 | rs6510259 | 3.74 | rs8100942 | 4.49 | 2.09 | 3.70E-02 |
| RP11-6N17.3 | 17 | rs9895985 | -2.59 | rs757351 | 3.57 | -2.08 | 3.77E-02 |
| PITRM1-AS1 | 10 | rs11596779 | 3.58 | rs2291103 | 4.95 | -2.08 | 3.78E-02 |
| RPL7L1 | 6 | rs7767888 | 2.58 | rs2894484 | -6.6 | -2.07 | 3.81E-02 |
| AC005702.4 | 17 | rs8075789 | -3.69 | rs2531899 | 6.73 | -2.07 | 3.83E-02 |
| ADIPOR1 | 1 | rs10920462 | 2.58 | rs4989513 | -6.26 | -2.07 | 3.83E-02 |
| POLB | 8 | rs1376442 | 2.92 | rs7002979 | -4.62 | -2.07 | 3.86E-02 |
| NDUFAF1 | 15 | rs28463309 | -2.64 | rs7168431 | -14.5 | 2.06 | 3.98E-02 |
| MRPS18AP1 | 3 | rs3895736 | 2.85 | rs1045482 | 10.67 | -2.05 | 4.00E-02 |
| SCYL3 | 1 | rs6663862 | -3.1 | rs688100 | -4.88 | 2.04 | 4.12E-02 |
| RP11-182J1.16 | 15 | rs12591597 | -2.33 | rs1107179 | -4.37 | -2.04 | 4.14E-02 |
| ALPK3 | 15 | rs8187736 | 2.58 | rs7183401 | -7.29 | 2.04 | 4.15E-02 |
| MEF2BNB | 19 | rs2238664 | 2.63 | rs1050544 | 4.06 | -2.04 | 4.18E-02 |
| KDM2B | 12 | rs7314454 | -2.81 | rs10849886 | -3.78 | -2.03 | 4.19E-02 |
| LCA5L | 21 | rs1888488 | -3.23 | rs9979289 | 8.75 | 2.03 | 4.19E-02 |
| HIBCH | 2 | rs3791767 | 2.17 | rs291431 | -11.08 | -2.03 | 4.25E-02 |
| RMDN3 | 15 | rs28602975 | -2.33 | rs4924490 | -6.24 | 2.02 | 4.30E-02 |
| ATIC | 2 | rs10498034 | 3.55 | rs16853834 | 7.61 | 2.02 | 4.33E-02 |
| RASA4DP | 7 | rs12534337 | -2.69 | rs4989124 | -6.1 | -2.02 | 4.33E-02 |
| AGA | 4 | rs2724754 | 3.15 | rs4690522 | -6.46 | -2.02 | 4.34E-02 |
| LSS | 21 | rs2839193 | -2.73 | rs2839193 | 5.09 | -2.01 | 4.44E-02 |
| FAM21C | 10 | rs2282350 | 2.64 | rs17159090 | -5.15 | 2.01 | 4.47E-02 |
| RAB7L1 | 1 | rs10900494 | 3.06 | rs823144 | -7.24 | -2.01 | 4.47E-02 |
| TMEM101 | 17 | rs1731902 | 2.74 | rs1731902 | -5.07 | -2.01 | 4.49E-02 |
| RP11-660L16.2 | 11 | rs11233933 | 2.82 | rs12791871 | -9.25 | -2 | 4.54E-02 |
| DNALI1 | 1 | rs17559127 | 3.18 | rs10908355 | 4.6 | 2 | 4.59E-02 |
| TOMM7 | 7 | rs2528850 | -2.88 | rs4722189 | -9.17 | -1.99 | 4.61E-02 |
| ZNF589 | 3 | rs3895736 | 2.85 | rs6796490 | 5.36 | -1.99 | 4.65E-02 |
| TMX3 | 18 | rs4891729 | -2.61 | rs1477990 | 4.31 | -1.99 | 4.66E-02 |
| TOR1A | 9 | rs7019254 | 2.73 | rs7873555 | -4.45 | -1.99 | 4.68E-02 |
| PEPD | 19 | rs4239577 | -3.43 | rs7247349 | -4.87 | -1.99 | 4.69E-02 |
| MEAF6 | 1 | rs17559127 | 3.18 | rs2273014 | 5.31 | -1.99 | 4.70E-02 |
| FDX1 | 11 | rs755128 | -3.67 | rs2358323 | 4.19 | -1.98 | 4.73E-02 |
| MIR3936 | 5 | rs1355095 | 2.89 | rs1007602 | -3.72 | -1.98 | 4.77E-02 |
| EP400NL | 12 | rs11147102 | -3.27 | rs12426418 | -5.39 | -1.97 | 4.83E-02 |
| RXRA | 9 | rs4842136 | 2.96 | rs11185647 | 5.96 | -1.97 | 4.88E-02 |
| GGT7 | 20 | rs932562 | -2.81 | rs12626122 | 4.89 | -1.97 | 4.90E-02 |
| RP11-304F15.3 | 17 | rs2197159 | 3.16 | rs2676801 | 5.13 | -1.97 | 4.90E-02 |
| RP11-540B6.6 | 15 | rs1524878 | -2.94 | rs11631814 | 4.87 | -1.97 | 4.93E-02 |
| EXOSC5 | 19 | rs12460494 | 2.86 | rs11670757 | 4.94 | -1.96 | 4.95E-02 |
| B4GALT1 | 9 | rs2481061 | -2.96 | rs10813959 | 7.43 | -1.96 | 4.96E-02 |
| C1QTNF4 | 11 | rs4752977 | -2.86 | rs3817334 | -7.21 | -1.96 | 4.96E-02 |
| HLA-AS1 | 1 | rs1577545 | 3.09 | rs903319 | 4.75 | 1.96 | 4.96E-02 |
