# Supplementary material for: Integrating transcriptome-wide study and mRNA expression profiles yields novel insights into the biological mechanism of chondropathies
Source: Arthritis Res Ther. 2019 Aug 27;21:194. doi: 10.1186/s13075-019-1978-8 (PMC6712880; doi:10.1186/s13075-019-1978-8)
Supplement: Supplementary file 4 — Table S4. TWAS identified significant genes in YBL for chondropathies. (DOCX 34 kb) [file 13075_2019_1978_MOESM4_ESM.docx]

Table S4 TWAS identified significant genes in YBL for chondropathies

| Gene | CHR | GWAS SNP | GWAS Z | EQTL SNP | EQTL Z | TWAS Z | TWAS P |
| --- | --- | --- | --- | --- | --- | --- | --- |
| RSRC1 | 3 | rs7624303 | -3.77 | rs2117764 | -13.64 | 3.88 | 1.03E-04 |
| NSA2 | 5 | rs1048167 | 3.69 | rs6877188 | 21.69 | 3.59 | 3.30E-04 |
| TPD52 | 8 | rs6473230 | -2.87 | rs2887723 | 10.96 | -3.57 | 3.62E-04 |
| CSF1R | 5 | rs2282806 | 4.1 | rs6874087 | 10.87 | -3.54 | 3.95E-04 |
| EXOSC2 | 9 | rs10751506 | -3.46 | rs10751506 | -8.17 | 3.45 | 5.69E-04 |
| HBP1 | 7 | rs711442 | 3.94 | rs2237659 | -9.24 | 3.34 | 8.33E-04 |
| MSC | 8 | rs6472675 | 2.9 | rs3779758 | 12.04 | 3.3 | 9.51E-04 |
| CDK5R1 | 17 | rs1018866 | 3.97 | rs7212466 | 19.51 | 3.23 | 1.22E-03 |
| CDC42SE2 | 5 | rs12153520 | 3.78 | rs3776019 | -8.81 | -3.19 | 1.42E-03 |
| SRGN | 10 | rs10998460 | -3.12 | rs2394525 | 6.02 | -3.18 | 1.46E-03 |
| FBXO33 | 14 | rs10872920 | 3.11 | rs10872918 | 3.58 | -3.15 | 1.64E-03 |
| NCK1 | 3 | rs6805715 | 3.94 | rs9682783 | -5.27 | 3.13 | 1.75E-03 |
| NME6 | 3 | rs3895736 | 2.85 | rs4312673 | 4.36 | 3.13 | 1.75E-03 |
| DDX20 | 1 | rs11102329 | 3.22 | rs499817 | 4.97 | 3.12 | 1.79E-03 |
| MTMR10 | 15 | rs1524878 | -2.94 | rs1524878 | -15.4 | 3.12 | 1.81E-03 |
| CPPED1 | 16 | rs938900 | -3.49 | rs1713478 | 6.21 | -3.07 | 2.13E-03 |
| TARS | 5 | rs4866357 | 3.24 | rs3736393 | -3.65 | -3.06 | 2.22E-03 |
| SLC38A7 | 16 | rs1437169 | -3.16 | rs1075567 | -5.95 | -3.04 | 2.33E-03 |
| FUT11 | 10 | rs16930753 | 3.27 | rs11000785 | -4.13 | 3.02 | 2.51E-03 |
| SCARB1 | 12 | rs4765138 | 2.85 | rs7954697 | 10.37 | 3.02 | 2.55E-03 |
| SNURF | 15 | rs11631911 | -3.05 | rs2732026 | 6.92 | -3.01 | 2.64E-03 |
| ANXA6 | 5 | rs2303028 | -3.46 | rs11750621 | 15.5 | -3.01 | 2.66E-03 |
| NUDT13 | 10 | rs16930753 | 3.27 | rs1332178 | -7.18 | -2.99 | 2.77E-03 |
| COG5 | 7 | rs711442 | 3.94 | rs11535285 | 22.85 | -2.99 | 2.82E-03 |
| EIF3F | 11 | rs11602553 | -3.54 | rs7941782 | 6.4 | -2.98 | 2.86E-03 |
| MTX3 | 5 | rs6859084 | -3.39 | rs383122 | -5.98 | -2.97 | 3.02E-03 |
| ETS1 | 11 | rs949101 | -3.51 | rs7127737 | 3.43 | 2.96 | 3.07E-03 |
| CAMK2D | 4 | rs13135212 | 2.9 | rs11936225 | -7.26 | 2.95 | 3.15E-03 |
| HSF2 | 6 | rs1367490 | -3.64 | rs573709 | -10.94 | 2.95 | 3.19E-03 |
| KCTD15 | 19 | rs4239577 | -3.43 | rs285684 | 7.6 | 2.94 | 3.26E-03 |
| RNASE6 | 14 | rs12586536 | 3.03 | rs4982347 | 16.93 | 2.93 | 3.38E-03 |
| SUPV3L1 | 10 | rs10998460 | -3.12 | rs12774307 | -8.03 | 2.91 | 3.59E-03 |
| FAM177A1 | 14 | rs10140560 | 3.83 | rs799488 | 9.82 | 2.9 | 3.75E-03 |
| CSNK1D | 17 | rs2306754 | -3.1 | rs3176835 | -8.27 | 2.9 | 3.78E-03 |
| MEGF9 | 9 | rs10985016 | -3.57 | rs2416760 | -15.7 | 2.89 | 3.85E-03 |
| SIGMAR1 | 9 | rs1571401 | -3.47 | rs12553321 | -16.06 | -2.89 | 3.89E-03 |
| TFPT | 19 | rs2114650 | 2.86 | rs16985367 | -12.87 | -2.88 | 3.99E-03 |
| PFKFB3 | 10 | rs11253886 | 3.17 | rs2516614 | 4.04 | -2.86 | 4.28E-03 |
| MRPS16 | 10 | rs16930753 | 3.27 | rs1064020 | 5.59 | 2.82 | 4.86E-03 |
| FAM149B1 | 10 | rs16930753 | 3.27 | rs12570678 | 5 | 2.79 | 5.30E-03 |
| SMPDL3A | 6 | rs1367490 | -3.64 | rs9401572 | -12.02 | 2.78 | 5.49E-03 |
| CENPK | 5 | rs33395 | 3.27 | rs42468 | 25.83 | 2.77 | 5.62E-03 |
| CGGBP1 | 3 | rs11128003 | 3.29 | rs12629581 | 9.19 | -2.76 | 5.75E-03 |
| NUP214 | 9 | rs10751506 | -3.46 | rs7033993 | 11.96 | 2.75 | 5.88E-03 |
| NDUFA10 | 2 | rs4854045 | 2.78 | rs8369 | -14.95 | 2.74 | 6.11E-03 |
| ZNF195 | 11 | rs2157763 | 3.11 | rs12417350 | -5.43 | -2.71 | 6.67E-03 |
| SH3GLB2 | 9 | rs10113912 | -2.58 | rs17455517 | -15.47 | 2.7 | 7.00E-03 |
| LYRM7 | 5 | rs12153520 | 3.78 | rs4706009 | 4.39 | 2.69 | 7.09E-03 |
| DFNA5 | 7 | rs2237318 | 2.87 | rs1476520 | 17.17 | 2.69 | 7.14E-03 |
| LILRB4 | 19 | rs891187 | 3.55 | rs370156 | 10.89 | 2.69 | 7.15E-03 |
| ANKIB1 | 7 | rs1989779 | 3.51 | rs403 | 6.85 | -2.67 | 7.51E-03 |
| ZNF721 | 4 | rs4130382 | 3.19 | rs13102358 | -9.88 | 2.67 | 7.65E-03 |
| SPOCK2 | 10 | rs1900515 | 2.58 | rs11000161 | -5.44 | 2.67 | 7.67E-03 |
| SFXN4 | 10 | rs3740558 | 3.06 | rs10749291 | 27.65 | 2.65 | 7.96E-03 |
| RASGRP1 | 15 | rs7496809 | 2.89 | rs12324402 | -7.85 | -2.64 | 8.27E-03 |
| KAT2B | 3 | rs9810613 | -3.63 | rs2948085 | 6.27 | 2.63 | 8.61E-03 |
| SCOC | 4 | rs358307 | 2.58 | rs358307 | 3.8 | 2.63 | 8.61E-03 |
| ARMC1 | 8 | rs4327888 | -3.65 | rs11995337 | 4.56 | -2.62 | 8.76E-03 |
| FBXO3 | 11 | rs831626 | 2.78 | rs12282096 | 5 | 2.62 | 8.84E-03 |
| RHOQ | 2 | rs9808496 | -4.2 | rs1868844 | 4.06 | -2.62 | 8.87E-03 |
| EFCAB4A | 11 | rs3924453 | 2.58 | rs11246300 | 8.52 | 2.62 | 8.92E-03 |
| ADIPOR1 | 1 | rs10920462 | 2.58 | rs2275737 | -6.96 | 2.61 | 9.15E-03 |
| WDYHV1 | 8 | rs4307325 | 3.28 | rs6999234 | -18.25 | -2.61 | 9.18E-03 |
| SAV1 | 14 | rs17780143 | -3.49 | rs10140023 | -6.17 | -2.61 | 9.18E-03 |
| ZNF525 | 19 | rs12610277 | 2.76 | rs7255193 | 9.34 | 2.6 | 9.36E-03 |
| 3-Mar | 5 | rs6892265 | -3.9 | rs9327428 | -9.16 | -2.6 | 9.37E-03 |
| ABHD15 | 17 | rs1017529 | 3.47 | rs497993 | 14.52 | -2.59 | 9.72E-03 |
| DHRS7 | 14 | rs1957309 | -2.58 | rs453730 | -7.98 | -2.58 | 9.77E-03 |
| PDCD2 | 6 | rs2180052 | 2.66 | rs4428484 | -4.39 | -2.58 | 9.99E-03 |
| ODC1 | 2 | rs1453084 | -3.31 | rs6432097 | -5.2 | 2.58 | 1.00E-02 |
| SNRPN | 15 | rs11631911 | -3.05 | rs2732026 | 8.07 | -2.58 | 1.00E-02 |
| KHK | 2 | rs7564363 | -3.03 | rs3769139 | 9.42 | -2.57 | 1.02E-02 |
| CMBL | 5 | rs17788857 | 2.58 | rs13180748 | 23.73 | 2.56 | 1.04E-02 |
| ALDH2 | 12 | rs12315146 | -2.17 | rs4767293 | 9.69 | 2.56 | 1.05E-02 |
| TREML2 | 6 | rs6926018 | -3.76 | rs9471490 | -6.98 | 2.55 | 1.07E-02 |
| FCN1 | 9 | rs4842136 | 2.96 | rs10120023 | 18.04 | 2.55 | 1.08E-02 |
| GHRL | 3 | rs696221 | -3.25 | rs12630739 | -11.06 | -2.55 | 1.09E-02 |
| SSH2 | 17 | rs12947084 | 3.64 | rs8073292 | -10.02 | -2.53 | 1.14E-02 |
| BLOC1S2 | 10 | rs7921931 | 2.83 | rs7073610 | 5.26 | 2.53 | 1.15E-02 |
| PHF19 | 9 | rs10985016 | -3.57 | rs10818482 | -7.48 | 2.52 | 1.18E-02 |
| HSF1 | 8 | rs4557742 | 2.73 | rs4977219 | -10.06 | -2.52 | 1.18E-02 |
| MBOAT2 | 2 | rs1006387 | 2.85 | rs6431975 | -4.49 | -2.5 | 1.24E-02 |
| SPTBN5 | 15 | rs28463309 | -2.64 | rs1672461 | -4.61 | -2.5 | 1.26E-02 |
| CLIP2 | 7 | rs2269081 | -2.17 | rs3135695 | -3.34 | 2.48 | 1.31E-02 |
| ADRB2 | 5 | rs2082382 | 2.7 | rs1801704 | 7.71 | 2.47 | 1.34E-02 |
| ANKLE2 | 12 | rs11147102 | -3.27 | rs13254 | 6.43 | -2.47 | 1.35E-02 |
| FAM83H | 8 | rs11136317 | -2.96 | rs28548729 | 4.72 | -2.46 | 1.39E-02 |
| FPGT | 1 | rs1601153 | -3.89 | rs11210443 | 7.39 | 2.46 | 1.40E-02 |
| ZHX2 | 8 | rs4871281 | -3.6 | rs7844465 | 16.79 | -2.45 | 1.44E-02 |
| AGA | 4 | rs2724754 | 3.15 | rs4690522 | -20.09 | -2.44 | 1.48E-02 |
| POLG2 | 17 | rs2302236 | -2.58 | rs17650301 | 4.58 | -2.44 | 1.48E-02 |
| NOXA1 | 9 | rs11145975 | -2.33 | rs10867091 | -7.12 | -2.44 | 1.49E-02 |
| LY6E | 8 | rs10283236 | -2.58 | rs10283236 | 9.59 | -2.43 | 1.50E-02 |
| CRK | 17 | rs7214541 | 3.48 | rs4239042 | -4.17 | -2.42 | 1.54E-02 |
| TOMM40L | 1 | rs3934593 | 2.76 | rs5082 | 5.94 | -2.42 | 1.55E-02 |
| CD300LB | 17 | rs3178300 | -2.58 | rs1699602 | 6.58 | -2.42 | 1.56E-02 |
| RCSD1 | 1 | rs11810018 | 4.14 | rs1933078 | 12.19 | 2.41 | 1.58E-02 |
| RBM26 | 13 | rs9545015 | 4.59 | rs2274554 | -5.55 | 2.41 | 1.59E-02 |
| CDCA4 | 14 | rs2033932 | -2.58 | rs3924892 | -13.25 | 2.41 | 1.61E-02 |
| VNN2 | 6 | rs531156 | -4.25 | rs1883613 | -22.03 | 2.41 | 1.62E-02 |
| CHURC1 | 14 | rs12884320 | 3.31 | rs4902336 | 29.38 | 2.4 | 1.63E-02 |
| CDCA7L | 7 | rs9639401 | 3.06 | rs7971 | -4.39 | 2.4 | 1.63E-02 |
| JSRP1 | 19 | rs12104154 | -2.58 | rs10415913 | 7.09 | -2.4 | 1.64E-02 |
| USP8 | 15 | rs7342574 | -2.58 | rs3098204 | 4.42 | 2.39 | 1.66E-02 |
| CSNK2A2 | 16 | rs1025067 | -2.81 | rs2242444 | 5.64 | -2.39 | 1.67E-02 |
| SRP19 | 5 | rs2545162 | 3.52 | rs2464803 | 3.77 | 2.39 | 1.70E-02 |
| GPD2 | 2 | rs297595 | -3.08 | rs298233 | 9.44 | -2.38 | 1.72E-02 |
| VIM | 10 | rs7914640 | 4.15 | rs359295 | -6.59 | 2.37 | 1.80E-02 |
| INTS6 | 13 | rs9568498 | 2.71 | rs9535661 | 9.9 | 2.37 | 1.80E-02 |
| ZNF410 | 14 | rs8019967 | 2.33 | rs8012476 | 3.31 | -2.36 | 1.82E-02 |
| SH2B3 | 12 | rs2188378 | -3.32 | rs688812 | -5.61 | 2.36 | 1.83E-02 |
| PTGER2 | 14 | rs999991 | 2.59 | rs1254600 | -11.85 | 2.36 | 1.84E-02 |
| PHLPP2 | 16 | rs16973286 | 3.08 | rs11539980 | -7.62 | 2.35 | 1.88E-02 |
| MFSD3 | 8 | rs4557742 | 2.73 | rs6981035 | -6.75 | 2.35 | 1.90E-02 |
| XRN2 | 20 | rs2424370 | -2.86 | rs6047413 | 27.71 | -2.34 | 1.92E-02 |
| LYSMD1 | 1 | rs11204764 | 2.58 | rs11807526 | 6.2 | -2.34 | 1.92E-02 |
| TRMT112 | 11 | rs1783811 | -2.65 | rs28395880 | -13.82 | 2.34 | 1.94E-02 |
| DCP2 | 5 | rs2545162 | 3.52 | rs13177101 | 14.3 | -2.33 | 1.99E-02 |
| SIPA1L2 | 1 | rs41482344 | 3.51 | rs7539735 | 15.15 | -2.33 | 2.00E-02 |
| BFSP1 | 20 | rs1987600 | 2.72 | rs6075218 | 4.44 | -2.31 | 2.08E-02 |
| SIRT4 | 12 | rs11065178 | -2.58 | rs16950058 | 12.27 | 2.31 | 2.08E-02 |
| CPT2 | 1 | rs1288516 | 3.43 | rs11578832 | -8.07 | -2.31 | 2.09E-02 |
| CLDN23 | 8 | rs1703974 | 2.58 | rs6994742 | 15.5 | 2.31 | 2.09E-02 |
| MFHAS1 | 8 | rs7018002 | 3.17 | rs17631052 | -5.59 | -2.29 | 2.18E-02 |
| LIN7A | 12 | rs12830912 | -2.93 | rs4842368 | -11.21 | 2.29 | 2.23E-02 |
| RAB6A | 11 | rs4944841 | 2.7 | rs1792199 | -8.41 | -2.28 | 2.23E-02 |
| BZW2 | 7 | rs2471907 | 3.07 | rs17625938 | -5.98 | -2.28 | 2.27E-02 |
| VCP | 9 | rs1571401 | -3.47 | rs679787 | -8.7 | -2.28 | 2.27E-02 |
| DUSP6 | 12 | rs922350 | -2.9 | rs1401881 | 9.93 | 2.28 | 2.28E-02 |
| RNF144B | 6 | rs4716220 | 2.17 | rs4716281 | 8.61 | 2.27 | 2.31E-02 |
| CDKN1B | 12 | rs10845716 | -3.65 | rs11055034 | -6.28 | -2.27 | 2.31E-02 |
| SESTD1 | 2 | rs2271761 | -2.98 | rs12622075 | 13.62 | -2.27 | 2.31E-02 |
| NUDT2 | 9 | rs1571401 | -3.47 | rs7039222 | 24.16 | 2.27 | 2.32E-02 |
| DUSP16 | 12 | rs17312912 | -3.47 | rs7972902 | -5.13 | -2.27 | 2.33E-02 |
| MYO1D | 17 | rs1018866 | 3.97 | rs17183295 | 5.57 | -2.27 | 2.34E-02 |
| HBG2 | 11 | rs4758435 | -2.84 | rs11036474 | 9.82 | -2.26 | 2.36E-02 |
| PRDM8 | 4 | rs11934116 | -3.03 | rs7664600 | -9.18 | 2.26 | 2.36E-02 |
| KDM5B | 1 | rs16849773 | -3.34 | rs10920462 | 6.75 | 2.26 | 2.38E-02 |
| TAF1B | 2 | rs4668664 | 2.61 | rs2245344 | -17.61 | -2.26 | 2.38E-02 |
| HEATR6 | 17 | rs8075789 | -3.69 | rs2087840 | 19.09 | -2.26 | 2.40E-02 |
| ISG15 | 1 | rs6690515 | -1.96 | rs6689813 | -2.95 | -2.25 | 2.43E-02 |
| DDX18 | 2 | rs11690896 | -3.5 | rs367201 | 13.7 | -2.25 | 2.45E-02 |
| ZNF683 | 1 | rs159528 | -3.44 | rs11247937 | -9.87 | 2.25 | 2.46E-02 |
| SLC26A6 | 3 | rs3895736 | 2.85 | rs9868809 | 3.17 | 2.25 | 2.47E-02 |
| ASAH1 | 8 | rs2720552 | 2.81 | rs7834136 | 8.21 | -2.25 | 2.47E-02 |
| DHRS9 | 2 | rs10490708 | -3.5 | rs7566044 | 17.93 | 2.25 | 2.47E-02 |
| SAMD4B | 19 | rs2074852 | 3.04 | rs11666780 | -4.72 | -2.25 | 2.47E-02 |
| MERTK | 2 | rs6710189 | 3.48 | rs6730521 | -13.82 | 2.24 | 2.50E-02 |
| MALT1 | 18 | rs8098336 | -2.7 | rs9944760 | -10.69 | 2.23 | 2.55E-02 |
| RAB12 | 18 | rs11659295 | 3.34 | rs1249489 | -3.38 | -2.23 | 2.55E-02 |
| SRP54 | 14 | rs10140560 | 3.83 | rs2273154 | -13.24 | 2.23 | 2.58E-02 |
| GFM1 | 3 | rs4680421 | -3.7 | rs6441215 | 13.85 | 2.23 | 2.60E-02 |
| AFF3 | 2 | rs934168 | 2.67 | rs6706188 | -7.87 | -2.22 | 2.62E-02 |
| COPG2 | 7 | rs10259462 | -2.94 | rs6467310 | -13.11 | -2.22 | 2.63E-02 |
| ISCU | 12 | rs741543 | 3.29 | rs888555 | 8.72 | 2.22 | 2.63E-02 |
| CUL1 | 7 | rs13242028 | -2.66 | rs3757441 | -3.44 | 2.22 | 2.65E-02 |
| GOLGA8A | 15 | rs11630666 | 3.64 | rs8039908 | -11.19 | 2.21 | 2.69E-02 |
| NRD1 | 1 | rs12134299 | -3.42 | rs2747525 | -13.25 | 2.21 | 2.71E-02 |
| ATP10A | 15 | rs8043094 | 2.64 | rs12908555 | 5.01 | 2.21 | 2.71E-02 |
| TSPAN15 | 10 | rs5030913 | -3.09 | rs2102339 | -3.88 | -2.2 | 2.75E-02 |
| ETV3 | 1 | rs1359647 | -2.89 | rs2231856 | -7.39 | 2.2 | 2.80E-02 |
| NSUN2 | 5 | rs9313172 | 3.6 | rs6887702 | 23.76 | -2.2 | 2.81E-02 |
| RPGRIP1 | 14 | rs2234636 | -2.74 | rs4981369 | -6.79 | 2.19 | 2.85E-02 |
| TRIM66 | 11 | rs11602553 | -3.54 | rs10840112 | 25.06 | 2.19 | 2.86E-02 |
| CORO7 | 16 | rs11862083 | 2.78 | rs7193204 | -8.71 | -2.19 | 2.88E-02 |
| PLAGL2 | 20 | rs6089119 | 2.58 | rs1205843 | 3.56 | -2.18 | 2.92E-02 |
| SLC37A1 | 21 | rs7281786 | 4.32 | rs1788414 | 4.12 | 2.18 | 2.94E-02 |
| ICAM2 | 17 | rs2302236 | -2.58 | rs3764868 | -6.26 | -2.18 | 2.96E-02 |
| POLRMT | 19 | rs3764572 | 3.24 | rs11672829 | -12.49 | 2.17 | 2.97E-02 |
| 2-Mar | 19 | rs11669207 | -2.85 | rs563320 | -9.96 | -2.17 | 2.98E-02 |
| METAP2 | 12 | rs12298894 | -2.58 | rs301009 | 8.47 | -2.17 | 3.02E-02 |
| METTL2B | 7 | rs10225988 | -2.77 | rs6467210 | 6.52 | -2.17 | 3.02E-02 |
| DOK4 | 16 | rs2001203 | -2.78 | rs223895 | 5.64 | 2.17 | 3.03E-02 |
| DPY19L2 | 12 | rs1828088 | 2.58 | rs12231958 | -6.78 | -2.16 | 3.06E-02 |
| METTL8 | 2 | rs4668380 | -2.99 | rs11674632 | -7.26 | 2.16 | 3.07E-02 |
| CEBPZ | 2 | rs10490665 | -2.71 | rs1158218 | -9.85 | -2.16 | 3.10E-02 |
| EFEMP2 | 11 | rs482320 | 2.33 | rs633800 | 10.67 | -2.15 | 3.13E-02 |
| DOCK10 | 2 | rs6750856 | -2.58 | rs7559750 | 9.4 | -2.15 | 3.17E-02 |
| PPP1R15A | 19 | rs2098684 | 2.88 | rs500079 | -14.67 | 2.14 | 3.21E-02 |
| 7-Mar | 2 | rs17495716 | 3.2 | rs7609477 | -4.46 | 2.14 | 3.22E-02 |
| TBXAS1 | 7 | rs4726603 | -3.52 | rs13760 | -10.34 | 2.14 | 3.24E-02 |
| MTFR1 | 8 | rs4327888 | -3.65 | rs7008671 | 11.36 | -2.13 | 3.30E-02 |
| S100P | 4 | rs6446523 | -3.03 | rs3822262 | -27.92 | 2.13 | 3.31E-02 |
| PAICS | 4 | rs1038115 | -3.27 | rs1520026 | -6.67 | -2.13 | 3.34E-02 |
| FLVCR2 | 14 | rs11622457 | 2.96 | rs3784003 | 12.41 | 2.13 | 3.34E-02 |
| SHKBP1 | 19 | rs814518 | 2.88 | rs2303729 | 11.48 | 2.12 | 3.36E-02 |
| ERCC3 | 2 | rs10803588 | 3.21 | rs4662713 | -7.46 | 2.12 | 3.38E-02 |
| COX4I1 | 16 | rs301163 | 3.29 | rs8587 | 5.14 | 2.12 | 3.40E-02 |
| CRIPT | 2 | rs9808496 | -4.2 | rs13385693 | 25.27 | -2.12 | 3.42E-02 |
| ZNF33B | 10 | rs3026713 | 3.16 | rs210280 | 7.27 | -2.12 | 3.44E-02 |
| PRDX1 | 1 | rs518216 | -2.68 | rs882803 | 10.3 | 2.11 | 3.45E-02 |
| YWHAH | 22 | rs2076054 | 3 | rs2858750 | 11.43 | 2.11 | 3.45E-02 |
| UBE2R2 | 9 | rs946847 | 2.86 | rs12375483 | 4.21 | -2.11 | 3.50E-02 |
| RAPGEFL1 | 17 | rs3934886 | -2.58 | rs9894845 | -4.67 | 2.11 | 3.51E-02 |
| ALDH16A1 | 19 | rs2303054 | -2.81 | rs10853810 | -9.2 | -2.11 | 3.52E-02 |
| EID1 | 15 | rs16961470 | 3.69 | rs10519197 | -13.91 | -2.11 | 3.52E-02 |
| ZYG11B | 1 | rs1288516 | 3.43 | rs4926930 | 16.71 | -2.1 | 3.55E-02 |
| FLII | 17 | rs16960870 | -2.33 | rs854774 | -5.8 | -2.1 | 3.58E-02 |
| CCNB1IP1 | 14 | rs12586536 | 3.03 | rs1713418 | -7.68 | 2.09 | 3.66E-02 |
| PSMD2 | 3 | rs1709621 | 2.58 | rs6845 | -16.88 | -2.09 | 3.70E-02 |
| ACVR2B | 3 | rs13085153 | -2.58 | rs7372545 | 3.64 | -2.08 | 3.73E-02 |
| DHFRL1 | 3 | rs6810199 | -2.33 | rs17749495 | 7.44 | -2.08 | 3.74E-02 |
| MAPKBP1 | 15 | rs28463309 | -2.64 | rs12902047 | -9.62 | -2.08 | 3.74E-02 |
| SEC62 | 3 | rs3732448 | 2.96 | rs3772185 | -6.22 | -2.08 | 3.75E-02 |
| DHTKD1 | 10 | rs1125791 | -2.9 | rs7894615 | 6.57 | -2.08 | 3.77E-02 |
| ATXN1L | 16 | rs16973286 | 3.08 | rs2335712 | 5.21 | 2.07 | 3.81E-02 |
| NDFIP1 | 5 | rs17097801 | 3.41 | rs12654522 | -6.17 | -2.07 | 3.82E-02 |
| ARIH2 | 3 | rs3895736 | 2.85 | rs4858828 | -3.84 | -2.07 | 3.83E-02 |
| SLC33A1 | 3 | rs17349490 | -3.07 | rs9837201 | -5.83 | 2.07 | 3.83E-02 |
| AACS | 12 | rs838869 | 2.71 | rs12228810 | -5 | 2.07 | 3.83E-02 |
| GCHFR | 15 | rs28602975 | -2.33 | rs7171945 | -8.12 | 2.07 | 3.87E-02 |
| AEN | 15 | rs6496512 | -2.9 | rs3743473 | 5.83 | -2.07 | 3.88E-02 |
| AP2B1 | 17 | rs8065286 | -3.02 | rs226085 | -5.43 | 2.07 | 3.89E-02 |
| HAL | 12 | rs7957408 | 3.1 | rs12307364 | -18.18 | 2.06 | 3.90E-02 |
| PID1 | 2 | rs7579884 | 3.17 | rs4972894 | -12.18 | -2.06 | 3.91E-02 |
| DNAJC27 | 2 | rs7601172 | -2.97 | rs1172293 | -7.99 | 2.06 | 3.92E-02 |
| VPS29 | 12 | rs2188378 | -3.32 | rs11829777 | -6.55 | 2.06 | 3.92E-02 |
| WDR6 | 3 | rs3895736 | 2.85 | rs7430198 | 17.04 | 2.06 | 3.94E-02 |
| ELOVL5 | 6 | rs11968088 | -2.64 | rs9474471 | 6.28 | -2.05 | 4.03E-02 |
| LIME1 | 20 | rs752606 | 3.52 | rs6011058 | 15.46 | -2.05 | 4.07E-02 |
| ACADM | 1 | rs1146644 | 2.87 | rs12123977 | 15.34 | -2.04 | 4.14E-02 |
| ESD | 13 | rs7996880 | -3.1 | rs1018578 | -7.85 | 2.04 | 4.15E-02 |
| BBS4 | 15 | rs4436752 | 3.13 | rs11637927 | 7.94 | -2.04 | 4.16E-02 |
| DHRS1 | 14 | rs10135546 | -2.71 | rs4568 | 13.36 | 2.03 | 4.22E-02 |
| ZNF613 | 19 | rs2163820 | -2.67 | rs12461478 | 10.84 | -2.03 | 4.22E-02 |
| PRKCD | 3 | rs4687657 | -3.02 | rs12490645 | 4.76 | -2.03 | 4.27E-02 |
| PIK3CG | 7 | rs6971719 | 2.92 | rs4730205 | 4.85 | 2.02 | 4.30E-02 |
| IER5 | 1 | rs2609480 | -2.84 | rs6686208 | 11.71 | 2.02 | 4.35E-02 |
| LPXN | 11 | rs12422108 | 2.58 | rs2515348 | -13.19 | -2.02 | 4.35E-02 |
| VPREB3 | 22 | rs2330625 | -2.58 | rs9612418 | 6.11 | 2.02 | 4.36E-02 |
| CCDC146 | 7 | rs3973061 | 2.58 | rs10488499 | -14.64 | -2.02 | 4.37E-02 |
| CHCHD5 | 2 | rs6756251 | 3.33 | rs768069 | -8.29 | 2.02 | 4.38E-02 |
| ATOX1 | 5 | rs11950215 | -3.21 | rs919262 | -10.94 | -2.01 | 4.40E-02 |
| WFS1 | 4 | rs12505016 | 3.18 | rs10002727 | -7.67 | 2.01 | 4.43E-02 |
| VENTX | 10 | rs2804004 | 2.58 | rs11101659 | -10.5 | 2.01 | 4.44E-02 |
| SLC12A7 | 5 | rs2962066 | -2.63 | rs10044441 | 5.46 | 2.01 | 4.45E-02 |
| HM13 | 20 | rs6088308 | 2.81 | rs6059958 | 11.44 | 2 | 4.51E-02 |
| ARIH1 | 15 | rs4436752 | 3.13 | rs7178141 | -8.46 | 2 | 4.52E-02 |
| SIRPB1 | 20 | rs4814138 | -2.58 | rs2250055 | 20.31 | 2 | 4.54E-02 |
| RBM47 | 4 | rs2136563 | 2.98 | rs11727143 | 8.83 | 2 | 4.56E-02 |
| FPR2 | 19 | rs2163820 | -2.67 | rs4802863 | -22.6 | 2 | 4.57E-02 |
| ROMO1 | 20 | rs2425049 | -2.85 | rs17426419 | 14.72 | -2 | 4.59E-02 |
| PSMC5 | 17 | rs2302236 | -2.58 | rs9914151 | -3.79 | -1.99 | 4.68E-02 |
| WIBG | 12 | rs1048103 | -2.72 | rs11171747 | 4.28 | 1.98 | 4.72E-02 |
| CECR1 | 22 | rs1125471 | 3.14 | rs5747018 | -5.58 | -1.98 | 4.73E-02 |
| LIX1L | 1 | rs16827018 | 3.17 | rs4471211 | -5.31 | -1.98 | 4.76E-02 |
| VAMP4 | 1 | rs6670432 | 3.21 | rs2298914 | 23.7 | -1.98 | 4.77E-02 |
| APPL2 | 12 | rs11112469 | 3.53 | rs1196781 | -15.22 | 1.97 | 4.90E-02 |
| P4HTM | 3 | rs3895736 | 2.85 | rs6446205 | -13.18 | -1.96 | 4.95E-02 |
| CIRH1A | 16 | rs7186053 | -3.35 | rs8061222 | -11.25 | 1.96 | 4.96E-02 |
| N6AMT2 | 13 | rs4770118 | 2.89 | rs17318109 | 4.21 | -1.96 | 4.98E-02 |
| GFI1 | 1 | rs17131598 | -2.87 | rs6662618 | -4.97 | 1.96 | 4.99E-02 |
