# Supplementary material for: Integrating transcriptome-wide study and mRNA expression profiles yields novel insights into the biological mechanism of chondropathies
Source: Arthritis Res Ther. 2019 Aug 27;21:194. doi: 10.1186/s13075-019-1978-8 (PMC6712880; doi:10.1186/s13075-019-1978-8)
Supplement: Supplementary file 5 — Table S5. Interative analyses identified candidate genes for knee OA. (DOCX 26 kb) [file 13075_2019_1978_MOESM5_ESM.docx]

Table S5 Interative analyses identified candidate genes for knee OA

| ID | Genes | CHR | GWAS | | mRNA expression | | EQTL | | | TWAS | | |
| --- | --- | --- | --- | --- | --- | --- | --- | --- | --- | --- | --- | --- |
|  |  |  | GWAS SNP | GWAS Z | P-value | FC | EQTL SNP | EQTL R^2^ | EQTL Z | Tissue | TWAS Z | TWASP |
| 1 | NSA2 | chr5 | rs1048167 | 3.69 | 2.12E-02 | -2.16 | rs6877188 | 0.61 | 14.78 | MS | 3.67 | 2.47E-04 |
|  |  |  | rs1048167 | 3.69 | 2.12E-02 | -2.16 | rs6877188 | 0.37 | 21.69 | YBL | 3.59 | 3.30E-04 |
| 2 | CSF1R | chr5 | rs2282806 | 4.1 | 2.83E-02 | 3.47 | rs6874087 | 0.07 | 10.87 | YBL | -3.54 | 3.95E-04 |
| 3 | MSC | chr8 | rs6472675 | 2.9 | 2.03E-02 | 4.41 | rs3779758 | 0.11 | 12.04 | YBL | 3.30 | 9.51E-04 |
| 4 | CDK5R1 | chr17 | rs1018866 | 3.97 | 4.71E-02 | 2.24 | rs7212466 | 0.29 | 19.51 | YBL | 3.23 | 1.22E-03 |
| 5 | MMP24 | chr20 | rs932562 | -2.81 | 2.64E-02 | 2.41 | rs6060341 | 0.08 | -5.76 | MS | 3.18 | 1.49E-03 |
| 6 | ARMC1 | chr8 | rs4327888 | -3.65 | 4.43E-02 | -2.64 | rs4737740 | 0.05 | -4.71 | MS | -3.15 | 1.62E-03 |
|  |  |  | rs4327888 | -3.65 | 4.43E-02 | -2.64 | rs11995337 | 0.01 | 4.56 | YBL | -2.62 | 8.76E-03 |
| 7 | NCK1 | chr3 | rs6805715 | 3.94 | 4.73E-02 | -2.26 | rs9682783 | 0.02 | -5.27 | YBL | 3.13 | 1.75E-03 |
| 8 | NME6 | chr3 | rs3895736 | 2.85 | 2.22E-03 | -2.93 | rs4312673 | 0.00 | 4.36 | YBL | 3.13 | 1.75E-03 |
|  |  |  | rs3895736 | 2.85 | 2.22E-03 | -2.93 | rs9819094 | 0.11 | -7.09 | MS | 2.58 | 9.93E-03 |
| 9 | DDX20 | chr1 | rs11102329 | 3.22 | 1.15E-02 | 2.69 | rs499817 | 0.01 | 4.97 | YBL | 3.12 | 1.79E-03 |
| 10 | SCARB1 | chr12 | rs4765138 | 2.85 | 6.75E-03 | -3.27 | rs7954697 | 0.08 | 10.37 | YBL | 3.02 | 2.55E-03 |
| 11 | ETS1 | chr11 | rs949101 | -3.51 | 2.36E-02 | 2.34 | rs7127737 | 0.00 | 3.43 | YBL | 2.96 | 3.07E-03 |
|  |  |  | rs949101 | -3.51 | 2.47E-02 | 2.17 | rs7127737 | 0.00 | 3.43 | YBL | 2.96 | 3.07E-03 |
| 12 | KCTD15 | chr19 | rs4239577 | -3.43 | 2.81E-05 | 2.94 | rs285684 | 0.03 | 7.6 | YBL | 2.94 | 3.26E-03 |
| 13 | FAM177A1 | chr14 | rs10140560 | 3.83 | 6.10E-03 | 3.96 | rs799488 | 0.07 | 9.82 | YBL | 2.90 | 3.75E-03 |
| 14 | CSNK1D | chr17 | rs2306754 | -3.1 | 2.44E-03 | -2.41 | rs3176835 | 0.04 | -8.27 | YBL | 2.90 | 3.78E-03 |
| 15 | PFKFB3 | chr10 | rs11253886 | 3.17 | 5.72E-04 | -4.46 | rs2516614 | 0.00 | 4.04 | YBL | -2.86 | 4.28E-03 |
| 16 | NDUFA10 | chr2 | rs4854045 | 2.78 | 3.18E-02 | -4.46 | rs8369 | 0.17 | -14.95 | YBL | 2.74 | 6.11E-03 |
|  |  |  | rs13393956 | 2.81 | 3.18E-02 | -4.46 | rs8369 | 0.05 | -6.28 | MS | 2.42 | 1.56E-02 |
| 17 | BCL7C | chr16 | rs34453065 | -3.58 | 7.32E-04 | -4.63 | rs17839549 | 0.09 | 6.55 | MS | -2.69 | 7.13E-03 |
| 18 | SPOCK2 | chr10 | rs1900515 | 2.58 | 1.00E-02 | 2.57 | rs11000161 | 0.02 | -5.44 | YBL | 2.67 | 7.67E-03 |
| 19 | SFXN4 | chr10 | rs3740558 | 3.06 | 4.50E-03 | -4.23 | rs10749291 | 0.61 | 27.65 | YBL | 2.65 | 7.96E-03 |
| 20 | SCOC | chr4 | rs358307 | 2.58 | 2.97E-03 | 3.13 | rs358307 | 0.01 | 3.8 | YBL | 2.63 | 8.61E-03 |
| 21 | RWDD1 | chr6 | rs7770769 | 3.17 | 5.01E-03 | -3.43 | rs2250263 | 0.03 | -4.72 | MS | -2.60 | 9.32E-03 |
| 22 | TCP11L1 | chr11 | rs3802789 | -2.58 | 2.63E-02 | 2.59 | rs7926030 | 0.21 | 8.91 | MS | -2.60 | 9.42E-03 |
| 23 | CDC25A | chr3 | rs3895736 | 2.85 | 1.81E-02 | 2.00 | rs13059037 | 0.25 | 9.64 | MS | -2.59 | 9.50E-03 |
| 24 | ABHD15 | chr17 | rs1017529 | 3.47 | 4.55E-02 | 2.14 | rs497993 | 0.17 | 14.52 | YBL | -2.59 | 9.72E-03 |
| 25 | DHRS7 | chr14 | rs1957309 | -2.58 | 2.59E-02 | -3.73 | rs453730 | 0.05 | -7.98 | YBL | -2.58 | 9.77E-03 |
| 26 | FNDC5 | chr1 | rs476153 | -3.06 | 1.15E-03 | 2.42 | rs785275 | 0.16 | 7.92 | MS | -2.58 | 9.88E-03 |
|  |  |  | rs476153 | -3.06 | 1.30E-02 | 2.23 | rs785275 | 0.16 | 7.92 | MS | -2.58 | 9.88E-03 |
| 27 | ODC1 | chr2 | rs1453084 | -3.31 | 1.50E-02 | -3.68 | rs6432097 | 0.02 | -5.2 | YBL | 2.58 | 1.00E-02 |
| 28 | SNRPN | chr15 | rs11631911 | -3.05 | 7.01E-04 | -4.46 | rs2732026 | 0.05 | 8.07 | YBL | -2.58 | 1.00E-02 |
|  |  |  | rs11631911 | -3.05 | 1.95E-03 | 2.30 | rs2732026 | 0.05 | 8.07 | YBL | -2.58 | 1.00E-02 |
| 29 | TRPT1 | chr11 | rs1783811 | -2.65 | 8.15E-03 | -2.54 | rs11603192 | 0.26 | 9.81 | MS | 2.55 | 1.06E-02 |
| 30 | HSF1 | chr8 | rs4557742 | 2.73 | 2.88E-02 | -2.00 | rs4977219 | 0.08 | -10.06 | YBL | -2.52 | 1.18E-02 |
| 31 | ACCS | chr11 | rs7111879 | 2.92 | 6.10E-03 | 4.74 | rs7951555 | 0.23 | 9.11 | MS | -2.50 | 1.26E-02 |
| 32 | C15orf40 | chr15 | rs4842993 | 3.46 | 1.77E-03 | 3.23 | rs1568657 | 0.06 | 5.67 | MS | 2.49 | 1.26E-02 |
| 33 | AGA | chr4 | rs2724754 | 3.15 | 4.91E-02 | -3.40 | rs4690522 | 0.30 | -20.09 | YBL | -2.44 | 1.48E-02 |
|  |  |  | rs2724754 | 3.15 | 4.91E-02 | -3.40 | rs4690522 | 0.11 | -6.46 | MS | -2.02 | 4.34E-02 |
| 34 | CRK | chr17 | rs7214541 | 3.48 | 1.63E-02 | -4.41 | rs4239042 | 0.01 | -4.17 | YBL | -2.42 | 1.54E-02 |
| 35 | CHURC1 | chr14 | rs12884320 | 3.31 | 3.70E-02 | -5.32 | rs2296327 | 0.18 | -8.24 | MS | -2.41 | 1.59E-02 |
|  |  |  | rs12884320 | 3.31 | 3.70E-02 | -5.32 | rs4902336 | 0.68 | 29.38 | YBL | 2.40 | 1.63E-02 |
| 36 | CSNK2A2 | chr16 | rs1025067 | -2.81 | 1.12E-02 | -2.81 | rs2242444 | 0.01 | 5.64 | YBL | -2.39 | 1.67E-02 |
| 37 | USP19 | chr3 | rs3895736 | 2.85 | 8.87E-03 | 2.92 | rs2286652 | 0.01 | -3.45 | MS | -2.37 | 1.76E-02 |
| 38 | AUH | chr9 | rs6479344 | 2.75 | 2.28E-02 | -2.51 | rs296646 | 0.01 | 3.84 | MS | 2.37 | 1.80E-02 |
| 39 | SESTD1 | chr2 | rs2271761 | -2.98 | 2.62E-02 | -3.27 | rs12622075 | 0.14 | 13.62 | YBL | -2.27 | 2.31E-02 |
| 40 | ANKRD10 | chr13 | rs2391890 | 3.01 | 2.30E-02 | -3.38 | rs2893386 | 0.01 | 4.17 | MS | -2.26 | 2.37E-02 |
| 41 | ABHD8 | chr19 | rs11879994 | 3.86 | 7.32E-03 | 2.47 | rs11086067 | 0.07 | -5.92 | MS | -2.25 | 2.41E-02 |
| 42 | DDX18 | chr2 | rs11690896 | -3.5 | 2.66E-02 | -2.30 | rs367201 | 0.14 | 13.7 | YBL | -2.25 | 2.45E-02 |
| 43 | DHRS9 | chr2 | rs10490708 | -3.5 | 2.51E-03 | 3.08 | rs7566044 | 0.25 | 17.93 | YBL | 2.25 | 2.47E-02 |
|  |  |  | rs10490708 | -3.5 | 2.42E-02 | 2.87 | rs7566044 | 0.25 | 17.93 | YBL | 2.25 | 2.47E-02 |
| 44 | COPG2 | chr7 | rs10259462 | -2.94 | 2.05E-02 | -2.69 | rs6467310 | 0.14 | -13.11 | YBL | -2.22 | 2.63E-02 |
| 45 | C9orf78 | chr9 | rs7019254 | 2.73 | 8.11E-03 | -3.02 | rs2274508 | 0.04 | -4.49 | MS | -2.21 | 2.68E-02 |
| 46 | PBLD | chr10 | rs12570981 | -3.04 | 3.19E-02 | 2.18 | rs34888891 | 0.10 | 6.88 | MS | -2.21 | 2.70E-02 |
| 47 | TSPAN15 | chr10 | rs5030913 | -3.09 | 2.58E-02 | 2.08 | rs2102339 | 0.00 | -3.88 | YBL | -2.20 | 2.75E-02 |
| 48 | PDCL3 | chr2 | rs1437968 | -2.65 | 2.38E-03 | -2.61 | rs13422218 | 0.08 | 6.11 | MS | 2.20 | 2.77E-02 |
| 49 | NSUN2 | chr5 | rs9313172 | 3.6 | 9.90E-04 | -6.76 | rs6887702 | 0.45 | 23.76 | YBL | -2.20 | 2.81E-02 |
| 50 | C15orf41 | chr15 | rs12899716 | -2.85 | 3.31E-02 | -2.37 | rs6495850 | 0.00 | 3.49 | MS | -2.19 | 2.83E-02 |
|  |  |  | rs12899716 | -2.85 | 3.05E-02 | 2.22 | rs6495850 | 0.00 | 3.49 | MS | -2.19 | 2.83E-02 |
| 51 | CORO7 | chr16 | rs11862083 | 2.78 | 4.18E-05 | 6.43 | rs7193204 | 0.06 | -8.71 | YBL | -2.19 | 2.88E-02 |
| 52 | CSTB | chr21 | rs2838280 | -3.19 | 9.41E-03 | -2.60 | rs9985006 | 0.14 | -7.94 | MS | -2.17 | 3.00E-02 |
| 53 | METAP2 | chr12 | rs12298894 | -2.58 | 2.09E-02 | -4.33 | rs301009 | 0.05 | 8.47 | YBL | -2.17 | 3.02E-02 |
| 54 | CEBPZ | chr2 | rs10490665 | -2.71 | 1.25E-02 | -2.98 | rs1158218 | 0.06 | -9.85 | YBL | -2.16 | 3.10E-02 |
| 55 | TBXAS1 | chr7 | rs4726603 | -3.52 | 5.38E-03 | 2.97 | rs13760 | 0.08 | -10.34 | YBL | 2.14 | 3.24E-02 |
| 56 | FGFRL1 | chr4 | rs4130382 | 3.19 | 2.01E-02 | 2.29 | rs34627176 | 0.08 | -5.7 | MS | 2.13 | 3.32E-02 |
| 57 | PAICS | chr4 | rs1038115 | -3.27 | 1.58E-03 | 3.61 | rs1520026 | 0.03 | -6.67 | YBL | -2.13 | 3.34E-02 |
| 58 | SHKBP1 | chr19 | rs814518 | 2.88 | 4.91E-03 | 2.03 | rs2303729 | 0.09 | 11.48 | YBL | 2.12 | 3.36E-02 |
| 59 | HAUS4 | chr14 | rs7469 | 2.91 | 2.13E-02 | -3.21 | rs11624528 | 0.08 | 5.85 | MS | 2.12 | 3.37E-02 |
| 60 | ERCC3 | chr2 | rs10803588 | 3.21 | 3.11E-02 | -3.41 | rs4662713 | 0.03 | -7.46 | YBL | 2.12 | 3.38E-02 |
| 61 | PRDX1 | chr1 | rs518216 | -2.68 | 2.40E-02 | 2.27 | rs882803 | 0.06 | 10.3 | YBL | 2.11 | 3.45E-02 |
| 62 | UBE2R2 | chr9 | rs946847 | 2.86 | 2.10E-03 | -3.83 | rs12375483 | 0.01 | 4.21 | YBL | -2.11 | 3.50E-02 |
| 63 | ALDH16A1 | chr19 | rs2303054 | -2.81 | 3.44E-02 | -3.76 | rs10853810 | 0.06 | -9.2 | YBL | -2.11 | 3.52E-02 |
| 64 | CCNB1IP1 | chr14 | rs12586536 | 3.03 | 2.02E-02 | 2.73 | rs1713418 | 0.04 | -7.68 | YBL | 2.09 | 3.66E-02 |
| 65 | RPL7L1 | chr6 | rs7767888 | 2.58 | 1.63E-02 | -4.04 | rs2894484 | 0.11 | -6.6 | MS | -2.07 | 3.81E-02 |
| 66 | GCHFR | chr15 | rs28602975 | -2.33 | 1.60E-05 | -8.99 | rs7171945 | 0.05 | -8.12 | YBL | 2.07 | 3.87E-02 |
| 67 | VPS29 | chr12 | rs2188378 | -3.32 | 5.48E-03 | -3.61 | rs11829777 | 0.03 | -6.55 | YBL | 2.06 | 3.92E-02 |
| 68 | NDUFAF1 | chr15 | rs28463309 | -2.64 | 3.08E-02 | -2.51 | rs7168431 | 0.56 | -14.5 | MS | 2.06 | 3.98E-02 |
| 69 | ESD | chr13 | rs7996880 | -3.1 | 4.35E-02 | -4.00 | rs1018578 | 0.05 | -7.85 | YBL | 2.04 | 4.15E-02 |
| 70 | DHRS1 | chr14 | rs10135546 | -2.71 | 3.98E-03 | 2.10 | rs4568 | 0.12 | 13.36 | YBL | 2.03 | 4.22E-02 |
| 71 | PRKCD | chr3 | rs4687657 | -3.02 | 3.19E-03 | -3.48 | rs12490645 | 0.02 | 4.76 | YBL | -2.03 | 4.27E-02 |
| 72 | VPREB3 | chr22 | rs2330625 | -2.58 | 4.37E-02 | 2.34 | rs9612418 | 0.03 | 6.11 | YBL | 2.02 | 4.36E-02 |
| 73 | RAB7L1 | chr1 | rs10900494 | 3.06 | 9.64E-03 | 2.14 | rs823144 | 0.14 | -7.24 | MS | -2.01 | 4.47E-02 |
| 74 | FAM21C | chr10 | rs2282350 | 2.64 | 1.19E-02 | 2.00 | rs17159090 | 0.06 | -5.15 | MS | 2.01 | 4.47E-02 |
| 75 | HM13 | chr20 | rs6088308 | 2.81 | 3.11E-03 | -2.68 | rs6059958 | 0.10 | 11.44 | YBL | 2.00 | 4.51E-02 |
|  |  |  | rs6088308 | 2.81 | 2.69E-02 | 2.38 | rs6059958 | 0.10 | 11.44 | YBL | 2.00 | 4.51E-02 |
| 76 | ARIH1 | chr15 | rs4436752 | 3.13 | 3.00E-02 | -2.24 | rs7178141 | 0.05 | -8.46 | YBL | 2.00 | 4.52E-02 |
| 77 | SIRPB1 | chr20 | rs4814138 | -2.58 | 7.98E-04 | 2.55 | rs2250055 | 0.32 | 20.31 | YBL | 2.00 | 4.54E-02 |
| 78 | TOMM7 | chr7 | rs2528850 | -2.88 | 8.93E-03 | 3.40 | rs4722189 | 0.23 | -9.17 | MS | -1.99 | 4.61E-02 |
| 79 | ZNF589 | chr3 | rs3895736 | 2.85 | 9.36E-03 | -3.21 | rs6796490 | 0.07 | 5.36 | MS | -1.99 | 4.65E-02 |
| 80 | MEAF6 | chr1 | rs17559127 | 3.18 | 2.21E-02 | -3.51 | rs2273014 | 0.07 | 5.31 | MS | -1.99 | 4.70E-02 |
| 81 | FDX1 | chr11 | rs755128 | -3.67 | 4.67E-03 | 3.38 | rs2358323 | 0.03 | 4.19 | MS | -1.98 | 4.73E-02 |
| 82 | CECR1 | chr22 | rs1125471 | 3.14 | 2.56E-02 | 2.37 | rs5747018 | 0.01 | -5.58 | YBL | -1.98 | 4.73E-02 |
| 83 | LIX1L | chr1 | rs16827018 | 3.17 | 1.44E-02 | -2.74 | rs4471211 | 0.01 | -5.31 | YBL | -1.98 | 4.76E-02 |
| 84 | C1QTNF4 | chr11 | rs4752977 | -2.86 | 2.94E-03 | -4.82 | rs3817334 | 0.14 | -7.21 | MS | -1.96 | 4.96E-02 |
