# Supplementary material for: Integrating transcriptome-wide study and mRNA expression profiles yields novel insights into the biological mechanism of chondropathies
Source: Arthritis Res Ther. 2019 Aug 27;21:194. doi: 10.1186/s13075-019-1978-8 (PMC6712880; doi:10.1186/s13075-019-1978-8)
Supplement: Supplementary file 6 — Table S6. Interative analyses identified causal genes for cartilage tumor. (DOCX 16 kb) [file 13075_2019_1978_MOESM6_ESM.docx]

Table S6 Interative analyses identified causal genes for cartilage tumor

| **ID** | **gene** | **CHR** | **GWAS data** | | **mRNA expression data** | | | | **EQTL data** | | | **TWAS data** | |
| --- | --- | --- | --- | --- | --- | --- | --- | --- | --- | --- | --- | --- | --- |
|  |  |  | **GWAS SNP** | **GWAS Z** | **PValue** | **logFC** | **FC** | **Tissue** | **EQTL SNP** | **EQTL R^2^** | **EQTL Z** | **TWAS Z** | **TWASP** |
| 1 | SRGN | 10 | rs10998460 | -3.12 | 4.18E-02 | 1.75 | 3.36 | YBL | rs2394525 | 0.01 | 6.02 | -3.18 | 1.46E-03 |
| 2 | ETS1 | 11 | rs949101 | -3.51 | 4.05E-05 | -1.11 | -2.16 | YBL | rs7127737 | 0.00 | 3.43 | 2.96 | 3.07E-03 |
| 3 | PFKFB3 | 10 | rs11253886 | 3.17 | 7.00E-03 | 1.87 | 3.66 | YBL | rs2516614 | 0.00 | 4.04 | -2.86 | 4.28E-03 |
| 4 | CHURC1 | 14 | rs12884320 | 3.31 | 1.36E-02 | -1.01 | -2.01 | MS | rs2296327 | 0.18 | -8.24 | -2.41 | 1.59E-02 |
|  |  |  | rs12884320 | 3.31 | 1.36E-02 | -1.01 | -2.01 | YBL | rs4902336 | 0.68 | 29.38 | 2.40 | 1.63E-02 |
| 5 | TRMT112 | 11 | rs1783811 | -2.65 | 1.01E-06 | -1.35 | -2.56 | YBL | rs28395880 | 0.15 | -13.82 | 2.34 | 1.94E-02 |
| 6 | PLEKHA1 | 10 | rs3763763 | 2.99 | 7.66E-05 | -1.39 | -2.62 | MS | rs4751890 | 0.04 | 4.55 | 2.27 | 2.29E-02 |
| 7 | AFF3 | 2 | rs934168 | 2.67 | 2.08E-04 | -1.23 | -2.35 | YBL | rs6706188 | 0.04 | -7.87 | -2.22 | 2.62E-02 |
| 8 | S100P | 4 | rs6446523 | -3.03 | 2.29E-02 | 2.46 | 5.49 | YBL | rs3822262 | 0.62 | -27.92 | 2.13 | 3.31E-02 |
| 9 | PSMC5 | 17 | rs2302236 | -2.58 | 4.21E-07 | -1.15 | -2.22 | YBL | rs9914151 | 0.00 | -3.79 | -1.99 | 4.68E-02 |
| 10 | C1QTNF4 | 11 | rs4752977 | -2.86 | 2.38E-05 | 1.73 | 3.32 | MS | rs3817334 | 0.14 | -7.21 | -1.96 | 4.96E-02 |
