# Supplementary material for: Integrating transcriptome-wide study and mRNA expression profiles yields novel insights into the biological mechanism of chondropathies
Source: Arthritis Res Ther. 2019 Aug 27;21:194. doi: 10.1186/s13075-019-1978-8 (PMC6712880; doi:10.1186/s13075-019-1978-8)
Supplement: Supplementary file 7 — Table S7. Interative analyses identified causal genes for spinal disc herniation. (DOCX 15 kb) [file 13075_2019_1978_MOESM7_ESM.docx]

Table S7 Interative analyses identified causal genes for spinal disc herniation

| **ID** | **Gene** | **CHR** | **GWAS** | | **mRNA expression** | | **EQTL** | | | **TWAS** | |
| --- | --- | --- | --- | --- | --- | --- | --- | --- | --- | --- | --- |
|  |  |  | **GWAS SNP** | **GWASZ** | **FC** | **Tissue** | **EQTL SNP** | **EQTLR2** | **EQTLZ** | **TWASZ** | **TWASP** |
| 1 | SUPV3L1 | 10 | rs10998460 | -3.12 | 3.22 | YBL | rs12774307 | 0.04 | -8.03 | 2.91 | 3.59E-03 |
| 2 | ZNF195 | 11 | rs2157763 | 3.11 | 2.25 | YBL | rs12417350 | 0.02 | -5.43 | -2.71 | 6.67E-03 |
| 3 | HBG2 | 11 | rs4758435 | -2.84 | 3.91 | YBL | rs11036474 | 0.07 | 9.82 | -2.26 | 2.36E-02 |
| 4 | VAMP4 | 1 | rs6670432 | 3.21 | 2.23 | YBL | rs2298914 | 0.44 | 23.7 | -1.98 | 4.77E-02 |
