# Supplementary material for: Integrating transcriptome-wide study and mRNA expression profiles yields novel insights into the biological mechanism of chondropathies
Source: Arthritis Res Ther. 2019 Aug 27;21:194. doi: 10.1186/s13075-019-1978-8 (PMC6712880; doi:10.1186/s13075-019-1978-8)
Supplement: Supplementary file 8 — Table S8. GO enrichment analyses results for knee OA. (DOCX 23 kb) [file 13075_2019_1978_MOESM8_ESM.docx]

Table S8 GO enrichment analyses results for knee OA

| **GeneSet** | **Tissue** | **Category** | **N** | **n** | ***P*** | **adjusted *P*** | **genes** |
| --- | --- | --- | --- | --- | --- | --- | --- |
| [GO_MATURATION_OF_LSU_RRNA](http://www.broadinstitute.org/gsea/msigdb/cards/GO_MATURATION_OF_LSU_RRNA) | MS | GO_BP | 13 | 2 | 1.18E-06 | 5.21E-03 | NSA2, RPL7L1 |
| [GO_NADH_DEHYDROGENASE_COMPLEX](http://www.broadinstitute.org/gsea/msigdb/cards/GO_NADH_DEHYDROGENASE_COMPLEX) | MS | GO_CC | 42 | 2 | 4.56E-05 | 1.84E-02 | NDUFAF1, NDUFA10 |
| [GO_MITOCHONDRIAL_PROTEIN_COMPLEX](http://www.broadinstitute.org/gsea/msigdb/cards/GO_MITOCHONDRIAL_PROTEIN_COMPLEX) | MS | GO_CC | 136 | 3 | 7.31E-05 | 1.84E-02 | NDUFAF1, NDUFA10, TOMM7 |
| [GO_MITOCHONDRIAL_MEMBRANE_PART](http://www.broadinstitute.org/gsea/msigdb/cards/GO_MITOCHONDRIAL_MEMBRANE_PART) | MS | GO_CC | 172 | 3 | 1.81E-04 | 2.10E-02 | NDUFAF1, NDUFA10, TOMM7 |
| [GO_RESPIRATORY_CHAIN](http://www.broadinstitute.org/gsea/msigdb/cards/GO_RESPIRATORY_CHAIN) | MS | GO_CC | 78 | 2 | 2.90E-04 | 2.81E-02 | NDUFAF1, NDUFA10 |
| [GO_OXIDOREDUCTASE_COMPLEX](http://www.broadinstitute.org/gsea/msigdb/cards/GO_OXIDOREDUCTASE_COMPLEX) | MS | GO_CC | 93 | 2 | 4.87E-04 | 3.18E-02 | NDUFAF1, NDUFA10 |
| [GO_INNER_MITOCHONDRIAL_MEMBRANE_PROTEIN_COMPLEX](http://www.broadinstitute.org/gsea/msigdb/cards/GO_INNER_MITOCHONDRIAL_MEMBRANE_PROTEIN_COMPLEX) | MS | GO_CC | 106 | 2 | 7.14E-04 | 3.45E-02 | NDUFAF1, NDUFA10 |
| [GO_PURINE_NUCLEOBASE_BIOSYNTHETIC_PROCESS](http://www.broadinstitute.org/gsea/msigdb/cards/GO_PURINE_NUCLEOBASE_BIOSYNTHETIC_PROCESS) | YBL | GO_BP | 12 | 2 | 4.75E-06 | 2.11E-02 | CECR1, PAICS |
| [GO_NUCLEOBASE_BIOSYNTHETIC_PROCESS](http://www.broadinstitute.org/gsea/msigdb/cards/GO_NUCLEOBASE_BIOSYNTHETIC_PROCESS) | YBL | GO_BP | 18 | 2 | 1.74E-05 | 3.86E-02 | CECR1, PAICS |
| [GO_PURINE_NUCLEOBASE_METABOLIC_PROCESS](http://www.broadinstitute.org/gsea/msigdb/cards/GO_PURINE_NUCLEOBASE_METABOLIC_PROCESS) | YBL | GO_BP | 21 | 2 | 2.82E-05 | 4.17E-02 | CECR1, PAICS |
| [GO_FC_GAMMA_RECEPTOR_SIGNALING_PATHWAY](http://www.broadinstitute.org/gsea/msigdb/cards/GO_FC_GAMMA_RECEPTOR_SIGNALING_PATHWAY) | YBL | GO_BP | 74 | 3 | 5.88E-05 | 4.88E-02 | CRK, PRKCD, NCK1 |
| [GO_PEPTIDYL_SERINE_MODIFICATION](http://www.broadinstitute.org/gsea/msigdb/cards/GO_PEPTIDYL_SERINE_MODIFICATION) | YBL | GO_BP | 148 | 4 | 6.37E-05 | 4.88E-02 | SPOCK2, CDK5R1, CSNK1D, PRKCD |
| [GO_ANDROGEN_METABOLIC_PROCESS](http://www.broadinstitute.org/gsea/msigdb/cards/GO_ANDROGEN_METABOLIC_PROCESS) | YBL | GO_BP | 29 | 2 | 7.62E-05 | 4.88E-02 | SCARB1, DHRS9 |
| [GO_PHAGOCYTOSIS](http://www.broadinstitute.org/gsea/msigdb/cards/GO_PHAGOCYTOSIS) | YBL | GO_BP | 160 | 4 | 9.21E-05 | 4.88E-02 | SCARB1, CRK, PRKCD, NCK1 |
| [GO_NEGATIVE_REGULATION_OF_MITOTIC_NUCLEAR_DIVISION](http://www.broadinstitute.org/gsea/msigdb/cards/GO_NEGATIVE_REGULATION_OF_MITOTIC_NUCLEAR_DIVISION) | YBL | GO_BP | 33 | 2 | 1.13E-04 | 4.88E-02 | CSNK2A2, NME6 |
| [GO_REGULATION_OF_CELLULAR_AMINE_METABOLIC_PROCESS](http://www.broadinstitute.org/gsea/msigdb/cards/GO_REGULATION_OF_CELLULAR_AMINE_METABOLIC_PROCESS) | YBL | GO_BP | 88 | 3 | 1.16E-04 | 4.88E-02 | SCARB1, ODC1, PRKCD |
| [GO_NUCLEOBASE_METABOLIC_PROCESS](http://www.broadinstitute.org/gsea/msigdb/cards/GO_NUCLEOBASE_METABOLIC_PROCESS) | YBL | GO_BP | 39 | 2 | 1.87E-04 | 4.88E-02 | CECR1, PAICS |
| [GO_REGULATION_OF_CATABOLIC_PROCESS](http://www.broadinstitute.org/gsea/msigdb/cards/GO_REGULATION_OF_CATABOLIC_PROCESS) | YBL | GO_BP | 728 | 8 | 1.99E-04 | 4.88E-02 | SCARB1, ARIH1, CSNK2A2, CDK5R1, CSNK1D, ODC1, PRKCD, SCOC |
| [GO_CELLULAR_RESPONSE_TO_REACTIVE_OXYGEN_SPECIES](http://www.broadinstitute.org/gsea/msigdb/cards/GO_CELLULAR_RESPONSE_TO_REACTIVE_OXYGEN_SPECIES) | YBL | GO_BP | 104 | 3 | 2.21E-04 | 4.88E-02 | PRDX1, ETS1, PRKCD |
| [GO_REGULATION_OF_ACTIN_FILAMENT_BASED_PROCESS](http://www.broadinstitute.org/gsea/msigdb/cards/GO_REGULATION_OF_ACTIN_FILAMENT_BASED_PROCESS) | YBL | GO_BP | 307 | 5 | 2.34E-04 | 4.88E-02 | CRK, CDK5R1, PRKCD, NCK1, CSF1R |
| [GO_RNA_SECONDARY_STRUCTURE_UNWINDING](http://www.broadinstitute.org/gsea/msigdb/cards/GO_RNA_SECONDARY_STRUCTURE_UNWINDING) | YBL | GO_BP | 43 | 2 | 2.50E-04 | 4.88E-02 | DDX20, DDX18 |
| [GO_NEGATIVE_REGULATION_OF_NUCLEAR_DIVISION](http://www.broadinstitute.org/gsea/msigdb/cards/GO_NEGATIVE_REGULATION_OF_NUCLEAR_DIVISION) | YBL | GO_BP | 45 | 2 | 2.86E-04 | 4.88E-02 | CSNK2A2, NME6 |
| [GO_PEPTIDYL_THREONINE_MODIFICATION](http://www.broadinstitute.org/gsea/msigdb/cards/GO_PEPTIDYL_THREONINE_MODIFICATION) | YBL | GO_BP | 46 | 2 | 3.06E-04 | 4.88E-02 | CDK5R1, PRKCD |
| [GO_PIGMENT_BIOSYNTHETIC_PROCESS](http://www.broadinstitute.org/gsea/msigdb/cards/GO_PIGMENT_BIOSYNTHETIC_PROCESS) | YBL | GO_BP | 47 | 2 | 3.26E-04 | 4.88E-02 | CECR1, PAICS |
| [GO_REGULATION_OF_NITRIC_OXIDE_SYNTHASE_ACTIVITY](http://www.broadinstitute.org/gsea/msigdb/cards/GO_REGULATION_OF_NITRIC_OXIDE_SYNTHASE_ACTIVITY) | YBL | GO_BP | 48 | 2 | 3.47E-04 | 4.88E-02 | SCARB1, GCHFR |
| [GO_REGULATION_OF_LIPID_CATABOLIC_PROCESS](http://www.broadinstitute.org/gsea/msigdb/cards/GO_REGULATION_OF_LIPID_CATABOLIC_PROCESS) | YBL | GO_BP | 52 | 2 | 4.40E-04 | 4.88E-02 | SCARB1, PRKCD |
| [GO_REGULATION_OF_LIPID_BIOSYNTHETIC_PROCESS](http://www.broadinstitute.org/gsea/msigdb/cards/GO_REGULATION_OF_LIPID_BIOSYNTHETIC_PROCESS) | YBL | GO_BP | 128 | 3 | 4.87E-04 | 4.88E-02 | DDX20, SCARB1, PRKCD |
| [GO_REGULATION_OF_CYTOSKELETON_ORGANIZATION](http://www.broadinstitute.org/gsea/msigdb/cards/GO_REGULATION_OF_CYTOSKELETON_ORGANIZATION) | YBL | GO_BP | 496 | 6 | 4.99E-04 | 4.88E-02 | CRK, CDK5R1, CSNK1D, PRKCD, NCK1, CSF1R |
| [GO_PHOSPHORYLATION](http://www.broadinstitute.org/gsea/msigdb/cards/GO_PHOSPHORYLATION) | YBL | GO_BP | 1218 | 10 | 5.58E-04 | 4.88E-02 | PFKFB3, CSNK2A2, CDK5R1, CSNK1D, ERCC3, NDUFA10, NME6, PRKCD, CSF1R, HSF1 |
| [GO_NEGATIVE_REGULATION_OF_CELL_DIVISION](http://www.broadinstitute.org/gsea/msigdb/cards/GO_NEGATIVE_REGULATION_OF_CELL_DIVISION) | YBL | GO_BP | 59 | 2 | 6.38E-04 | 4.88E-02 | CSNK2A2, NME6 |
| [GO_REGULATION_OF_MONOOXYGENASE_ACTIVITY](http://www.broadinstitute.org/gsea/msigdb/cards/GO_REGULATION_OF_MONOOXYGENASE_ACTIVITY) | YBL | GO_BP | 59 | 2 | 6.38E-04 | 4.88E-02 | SCARB1, GCHFR |
| [GO_PURINE_CONTAINING_COMPOUND_BIOSYNTHETIC_PROCESS](http://www.broadinstitute.org/gsea/msigdb/cards/GO_PURINE_CONTAINING_COMPOUND_BIOSYNTHETIC_PROCESS) | YBL | GO_BP | 138 | 3 | 6.46E-04 | 4.88E-02 | CECR1, NME6, PAICS |
| [GO_NUCLEOTIDE_PHOSPHORYLATION](http://www.broadinstitute.org/gsea/msigdb/cards/GO_NUCLEOTIDE_PHOSPHORYLATION) | YBL | GO_BP | 60 | 2 | 6.70E-04 | 4.88E-02 | PFKFB3, NME6 |
| [GO_REGULATION_OF_CELLULAR_RESPONSE_TO_STRESS](http://www.broadinstitute.org/gsea/msigdb/cards/GO_REGULATION_OF_CELLULAR_RESPONSE_TO_STRESS) | YBL | GO_BP | 688 | 7 | 6.99E-04 | 4.88E-02 | PRDX1, CSNK2A2, CDK5R1, PRKCD, NCK1, SCOC, HSF1 |
| [GO_REGULATION_OF_PHOSPHOLIPID_METABOLIC_PROCESS](http://www.broadinstitute.org/gsea/msigdb/cards/GO_REGULATION_OF_PHOSPHOLIPID_METABOLIC_PROCESS) | YBL | GO_BP | 61 | 2 | 7.04E-04 | 4.88E-02 | SCARB1, PRKCD |
| [GO_CELLULAR_RESPONSE_TO_HYDROGEN_PEROXIDE](http://www.broadinstitute.org/gsea/msigdb/cards/GO_CELLULAR_RESPONSE_TO_HYDROGEN_PEROXIDE) | YBL | GO_BP | 61 | 2 | 7.04E-04 | 4.88E-02 | ETS1, PRKCD |
| [GO_PIGMENT_METABOLIC_PROCESS](http://www.broadinstitute.org/gsea/msigdb/cards/GO_PIGMENT_METABOLIC_PROCESS) | YBL | GO_BP | 62 | 2 | 7.38E-04 | 4.88E-02 | CECR1, PAICS |
| [GO_OXIDATION_REDUCTION_PROCESS](http://www.broadinstitute.org/gsea/msigdb/cards/GO_OXIDATION_REDUCTION_PROCESS) | YBL | GO_BP | 875 | 8 | 7.62E-04 | 4.88E-02 | PRDX1, PFKFB3, DHRS1, DHRS7, ALDH16A1, DHRS9, NDUFA10, TBXAS1 |
| [GO_REGULATION_OF_PROTEASOMAL_UBIQUITIN_DEPENDENT_PROTEIN_CATABOLIC_PROCESS](http://www.broadinstitute.org/gsea/msigdb/cards/GO_REGULATION_OF_PROTEASOMAL_UBIQUITIN_DEPENDENT_PROTEIN_CATABOLIC_PROCESS) | YBL | GO_BP | 147 | 3 | 8.19E-04 | 4.88E-02 | ARIH1, CSNK2A2, CSNK1D |
| [GO_PURINE_CONTAINING_COMPOUND_METABOLIC_PROCESS](http://www.broadinstitute.org/gsea/msigdb/cards/GO_PURINE_CONTAINING_COMPOUND_METABOLIC_PROCESS) | YBL | GO_BP | 392 | 5 | 8.51E-04 | 4.88E-02 | PFKFB3, NDUFA10, CECR1, NME6, PAICS |
| [GO_MEIOTIC_CELL_CYCLE_PROCESS](http://www.broadinstitute.org/gsea/msigdb/cards/GO_MEIOTIC_CELL_CYCLE_PROCESS) | YBL | GO_BP | 149 | 3 | 8.61E-04 | 4.88E-02 | CCNB1IP1, NSUN2, HSF1 |
| [GO_PROTEIN_POLYMERIZATION](http://www.broadinstitute.org/gsea/msigdb/cards/GO_PROTEIN_POLYMERIZATION) | YBL | GO_BP | 66 | 2 | 8.86E-04 | 4.88E-02 | CORO7, CSNK1D |
| [GO_POSITIVE_REGULATION_OF_LIPID_BIOSYNTHETIC_PROCESS](http://www.broadinstitute.org/gsea/msigdb/cards/GO_POSITIVE_REGULATION_OF_LIPID_BIOSYNTHETIC_PROCESS) | YBL | GO_BP | 66 | 2 | 8.86E-04 | 4.88E-02 | SCARB1, PRKCD |
| [GO_PROTEIN_MATURATION](http://www.broadinstitute.org/gsea/msigdb/cards/GO_PROTEIN_MATURATION) | YBL | GO_BP | 262 | 4 | 8.92E-04 | 4.88E-02 | TSPAN15, METAP2, HM13, AGA |
| [GO_POSITIVE_REGULATION_OF_ENDOTHELIAL_CELL_MIGRATION](http://www.broadinstitute.org/gsea/msigdb/cards/GO_POSITIVE_REGULATION_OF_ENDOTHELIAL_CELL_MIGRATION) | YBL | GO_BP | 67 | 2 | 9.25E-04 | 4.88E-02 | ETS1, SCARB1 |
| [GO_GOLGI_ASSOCIATED_VESICLE_MEMBRANE](http://www.broadinstitute.org/gsea/msigdb/cards/GO_GOLGI_ASSOCIATED_VESICLE_MEMBRANE) | YBL | GO_CC | 49 | 2 | 3.69E-04 | 4.38E-02 | HM13, COPG2 |
| [GO_GOLGI_ASSOCIATED_VESICLE](http://www.broadinstitute.org/gsea/msigdb/cards/GO_GOLGI_ASSOCIATED_VESICLE) | YBL | GO_CC | 81 | 2 | 1.60E-03 | 4.48E-02 | HM13, COPG2 |
| [GO_TRANS_GOLGI_NETWORK](http://www.broadinstitute.org/gsea/msigdb/cards/GO_TRANS_GOLGI_NETWORK) | YBL | GO_CC | 190 | 3 | 2.11E-03 | 4.89E-02 | CORO7, NCK1, SCOC |
| [GO_1_PHOSPHATIDYLINOSITOL_BINDING](http://www.broadinstitute.org/gsea/msigdb/cards/GO_1_PHOSPHATIDYLINOSITOL_BINDING) | YBL | GO_MF | 19 | 2 | 2.06E-05 | 1.86E-02 | SCARB1, SESTD1 |
| [GO_EPHRIN_RECEPTOR_BINDING](http://www.broadinstitute.org/gsea/msigdb/cards/GO_EPHRIN_RECEPTOR_BINDING) | YBL | GO_MF | 24 | 2 | 4.26E-05 | 1.92E-02 | CRK, CDK5R1 |
| [GO_PHOSPHATIDYLSERINE_BINDING](http://www.broadinstitute.org/gsea/msigdb/cards/GO_PHOSPHATIDYLSERINE_BINDING) | YBL | GO_MF | 33 | 2 | 1.13E-04 | 2.72E-02 | SCARB1, SESTD1 |
| [GO_CARBOXY_LYASE_ACTIVITY](http://www.broadinstitute.org/gsea/msigdb/cards/GO_CARBOXY_LYASE_ACTIVITY) | YBL | GO_MF | 35 | 2 | 1.35E-04 | 2.72E-02 | ODC1, PAICS |
| [GO_PURINE_NTP_DEPENDENT_HELICASE_ACTIVITY](http://www.broadinstitute.org/gsea/msigdb/cards/GO_PURINE_NTP_DEPENDENT_HELICASE_ACTIVITY) | YBL | GO_MF | 96 | 3 | 1.62E-04 | 2.72E-02 | DDX20, DDX18, ERCC3 |
| [GO_AMINO_ACID_BINDING](http://www.broadinstitute.org/gsea/msigdb/cards/GO_AMINO_ACID_BINDING) | YBL | GO_MF | 107 | 3 | 2.46E-04 | 2.72E-02 | SCARB1, GCHFR, SESTD1 |
| [GO_CARBON_CARBON_LYASE_ACTIVITY](http://www.broadinstitute.org/gsea/msigdb/cards/GO_CARBON_CARBON_LYASE_ACTIVITY) | YBL | GO_MF | 50 | 2 | 3.92E-04 | 2.72E-02 | ODC1, PAICS |
| [GO_SH3_SH2_ADAPTOR_ACTIVITY](http://www.broadinstitute.org/gsea/msigdb/cards/GO_SH3_SH2_ADAPTOR_ACTIVITY) | YBL | GO_MF | 52 | 2 | 4.40E-04 | 2.72E-02 | CRK, NCK1 |
| [GO_KINASE_ACTIVITY](http://www.broadinstitute.org/gsea/msigdb/cards/GO_KINASE_ACTIVITY) | YBL | GO_MF | 834 | 8 | 5.40E-04 | 2.72E-02 | PFKFB3, CSNK2A2, CDK5R1, CSNK1D, ERCC3, NME6, PRKCD, CSF1R |
| [GO_PHOSPHOPROTEIN_BINDING](http://www.broadinstitute.org/gsea/msigdb/cards/GO_PHOSPHOPROTEIN_BINDING) | YBL | GO_MF | 60 | 2 | 6.70E-04 | 2.72E-02 | CRK, CSNK1D |
| [GO_OXIDOREDUCTASE_ACTIVITY](http://www.broadinstitute.org/gsea/msigdb/cards/GO_OXIDOREDUCTASE_ACTIVITY) | YBL | GO_MF | 697 | 7 | 7.60E-04 | 2.72E-02 | PRDX1, DHRS1, DHRS7, ALDH16A1, DHRS9, NDUFA10, TBXAS1 |
| [GO_ADENYL_NUCLEOTIDE_BINDING](http://www.broadinstitute.org/gsea/msigdb/cards/GO_ADENYL_NUCLEOTIDE_BINDING) | YBL | GO_MF | 1490 | 11 | 8.41E-04 | 2.72E-02 | DDX20, PFKFB3, CSNK2A2, CSNK1D, DDX18, ERCC3, NME6, PRKCD, PAICS, CSF1R, UBE2R2 |
| [GO_MODIFIED_AMINO_ACID_BINDING](http://www.broadinstitute.org/gsea/msigdb/cards/GO_MODIFIED_AMINO_ACID_BINDING) | YBL | GO_MF | 65 | 2 | 8.47E-04 | 2.72E-02 | SCARB1, SESTD1 |
| [GO_RNA_HELICASE_ACTIVITY](http://www.broadinstitute.org/gsea/msigdb/cards/GO_RNA_HELICASE_ACTIVITY) | YBL | GO_MF | 66 | 2 | 8.86E-04 | 2.72E-02 | DDX20, DDX18 |
| [GO_HELICASE_ACTIVITY](http://www.broadinstitute.org/gsea/msigdb/cards/GO_HELICASE_ACTIVITY) | YBL | GO_MF | 151 | 3 | 9.05E-04 | 2.72E-02 | DDX20, DDX18, ERCC3 |
| [GO_SIGNALING_ADAPTOR_ACTIVITY](http://www.broadinstitute.org/gsea/msigdb/cards/GO_SIGNALING_ADAPTOR_ACTIVITY) | YBL | GO_MF | 74 | 2 | 1.23E-03 | 3.01E-02 | CRK, NCK1 |
| [GO_BINDING_BRIDGING](http://www.broadinstitute.org/gsea/msigdb/cards/GO_BINDING_BRIDGING) | YBL | GO_MF | 172 | 3 | 1.46E-03 | 3.01E-02 | DDX20, CRK, NCK1 |
| [GO_PROTEIN_SERINE_THREONINE_KINASE_ACTIVITY](http://www.broadinstitute.org/gsea/msigdb/cards/GO_PROTEIN_SERINE_THREONINE_KINASE_ACTIVITY) | YBL | GO_MF | 443 | 5 | 1.59E-03 | 3.11E-02 | CSNK2A2, CDK5R1, CSNK1D, ERCC3, PRKCD |
| [GO_TRANSFERASE_ACTIVITY_TRANSFERRING_PHOSPHORUS_CONTAINING_GROUPS](http://www.broadinstitute.org/gsea/msigdb/cards/GO_TRANSFERASE_ACTIVITY_TRANSFERRING_PHOSPHORUS_CONTAINING_GROUPS) | YBL | GO_MF | 978 | 8 | 1.66E-03 | 3.12E-02 | PFKFB3, CSNK2A2, CDK5R1, CSNK1D, ERCC3, NME6, PRKCD, CSF1R |
| [GO_PROTEIN_KINASE_ACTIVITY](http://www.broadinstitute.org/gsea/msigdb/cards/GO_PROTEIN_KINASE_ACTIVITY) | YBL | GO_MF | 638 | 6 | 2.15E-03 | 3.46E-02 | CSNK2A2, CDK5R1, CSNK1D, ERCC3, PRKCD, CSF1R |
| [GO_UBIQUITIN_LIKE_PROTEIN_LIGASE_ACTIVITY](http://www.broadinstitute.org/gsea/msigdb/cards/GO_UBIQUITIN_LIKE_PROTEIN_LIGASE_ACTIVITY) | YBL | GO_MF | 197 | 3 | 2.40E-03 | 3.80E-02 | CCNB1IP1, ARIH1, UBE2R2 |
| [GO_ORGANIC_ACID_BINDING](http://www.broadinstitute.org/gsea/msigdb/cards/GO_ORGANIC_ACID_BINDING) | YBL | GO_MF | 207 | 3 | 2.87E-03 | 4.22E-02 | SCARB1, GCHFR, SESTD1 |
| [GO_ENZYME_BINDING](http://www.broadinstitute.org/gsea/msigdb/cards/GO_ENZYME_BINDING) | YBL | GO_MF | 1726 | 11 | 2.96E-03 | 4.22E-02 | DDX20, TSPAN15, ETS1, GCHFR, ARIH1, CDK5R1, HM13, PRKCD, NCK1, CSF1R, UBE2R2 |
| [GO_GLYCOPROTEIN_BINDING](http://www.broadinstitute.org/gsea/msigdb/cards/GO_GLYCOPROTEIN_BINDING) | YBL | GO_MF | 101 | 2 | 3.01E-03 | 4.22E-02 | CSNK1D, CECR1 |
| [GO_PROTEIN_N_TERMINUS_BINDING](http://www.broadinstitute.org/gsea/msigdb/cards/GO_PROTEIN_N_TERMINUS_BINDING) | YBL | GO_MF | 103 | 2 | 3.18E-03 | 4.27E-02 | CSNK2A2, ERCC3 |

**Note:** MS stands for muscle skeleton.YBL stands for peripheral blood. GO_BP, GO_CC,and GO_MF mean GO_biological process, GO_cellular component and GO_molecular function separately. N means the number of the enriched genes in the GO analysis, and n means the number of genes participating in the GO enrichment analysis in our study.
